# Distinct origin and region-dependent contribution of stromal fibroblasts to fibrosis following traumatic injury in mice

**DOI:** 10.1038/s41593-024-01678-4

**Published:** 2024-06-07

**Authors:** Daniel Holl, Wing Fung Hau, Anais Julien, Shervin Banitalebi, Jannis Kalkitsas, Soniya Savant, Enric Llorens-Bobadilla, Yann Herault, Guillaume Pavlovic, Mahmood Amiry-Moghaddam, David Oliveira Dias, Christian Göritz

**Affiliations:** 1 Department of Cell and Molecular Biology, Karolinska Institutet, Stockholm, Sweden; 2 Center for Neuromusculoskeletal Restorative Medicine, Shatin, Hong Kong; 3 Division of Anatomy, Department of Molecular Medicine, Institute of Basic Medical Sciences, University of Oslo, Oslo, Norway; 4 Université de Strasbourg, CNRS, INSERM, Institut de Génétique Biologie Moléculaire et Cellulaire (IGBMC), Illkirch, France; 5 Université de Strasbourg, CNRS, INSERM, CELPHEDIA, PHENOMIN-Institut Clinique de la Souris, Illkirch-Graffenstaden, France

**Keywords:** Spinal cord injury, Neuro-vascular interactions

## Abstract

Fibrotic scar tissue formation occurs in humans and mice. The fibrotic scar impairs tissue regeneration and functional recovery. However, the origin of scar-forming fibroblasts is unclear. Here, we show that stromal fibroblasts forming the fibrotic scar derive from two populations of perivascular cells after spinal cord injury (SCI) in adult mice of both sexes. We anatomically and transcriptionally identify the two cell populations as pericytes and perivascular fibroblasts. Fibroblasts and pericytes are enriched in the white and gray matter regions of the spinal cord, respectively. Both cell populations are recruited in response to SCI and inflammation. However, their contribution to fibrotic scar tissue depends on the location of the lesion. Upon injury, pericytes and perivascular fibroblasts become activated and transcriptionally converge on the generation of stromal myofibroblasts. Our results show that pericytes and perivascular fibroblasts contribute to the fibrotic scar in a region-dependent manner.

## Abstract (web summary)

The origin and composition of stromal fibroblasts in the fibrotic CNS scar are unclear. Here, the authors demonstrate that pericytes and perivascular fibroblasts contribute to fibrotic scarring following spinal cord injury in mice in a region-dependent manner.

## Main

Regeneration in the adult mammalian central nervous system (CNS) is limited. One major limiting factor is the formation of fibrotic scar tissue^1–4^. Initially, the recruitment of fibroblasts and deposition of fibrotic extracellular matrix (ECM) are required for wound closure, but they consequently result in the formation of persistent scar tissue^3,5,6^. Importantly, a moderate reduction of fibrotic scarring, which still prompts wound closure, promotes axon regeneration and functional recovery after spinal cord injury (SCI)^1,3,7–9^ and alleviates disease severity in experimental autoimmune encephalomyelitis (EAE)^4^. These findings suggest fibrotic scarring as a therapeutic target to improve regeneration after CNS lesions. Understanding the identity of scar-forming cells and fibrotic tissue formation in more detail may allow targeting the scar-forming process therapeutically.

Using in vivo lineage tracing, we previously identified a small population of glutamate aspartate transporter (GLAST; gene name also known as *Slc1a3*)-expressing perivascular cells, named type A pericytes, as the cellular origin of scar-forming fibroblasts throughout the CNS in response to autoimmune demyelinating disease and traumatic and ischemic lesions^6,10^. GLAST^+^ cells represent roughly 10% of all platelet-derived growth factor receptor β (PDGFRβ)^+^ perivascular cells in the uninjured adult mouse brain and spinal cord^6,10^. Similarly, a subset of perivascular cells expressing GLAST and PDGFRβ can be found in the healthy human brain and spinal cord^10^. Single-cell transcriptomic studies of vascular cells in the mouse brain have defined perivascular fibroblasts^11^, which share *Pdgfrb* expression with pericytes but can be distinguished by the expression of *Pdgfra* and *Col1a1* (refs. ^4,11^). Based on a *Col1a1*-green fluorescent protein (GFP) reporter line, which reflects *Col1a1* transcriptional activity, perivascular fibroblasts have been suggested as the origin of fibrotic scar tissue after SCI and EAE^4,12^. Direct lineage-tracing evidence was limited to *Col1a2*-expressing cells^4^ after EAE, which, in addition to perivascular fibroblasts, include some pericytes and vascular smooth muscle cells (vSMCs) in the CNS^11^. Perivascular fibroblasts reside in the Virchow–Robin space along penetrating arterioles and large ascending venules in an abluminal position to vSMCs^4,13^. In contrast, pericytes are found in the microvasculature, embedded in the vascular basement membrane^14^. Furthermore, pericytes and fibroblasts show distinct gene expression profiles at the single-cell level, allowing them to be distinguished from one another^11^.

Based on single-cell RNA sequencing and lineage tracing in the context of SCI, we confined the fibrotic scar-forming capacity to a specific subset of GLAST-expressing pericytes and perivascular fibroblasts. Both cell populations are recruited locally in response to injury and inflammation. Given their anatomical location, pericytes predominantly contribute to focal lesions in the gray matter, whereas perivascular fibroblasts contribute more extensively to focal white matter lesions. Transcriptionally, we discerned five stromal cell populations at the lesion site, revealing functional diversity in fibrotic ECM deposition, revascularization and immune regulation. Furthermore, we reconstructed the molecular trajectories leading to fibrotic scar formation from pericytes and perivascular fibroblasts after SCI, demonstrating their convergence in generating stromal myofibroblasts. Our findings unveil previously unrecognized heterogeneity in the origin of fibrotic scar tissue, expanding our current understanding of the complexity underlying CNS scar formation^4,15^.

## Results

### Fibrotic scar tissue-forming perivascular cells

**Fig. 1:**
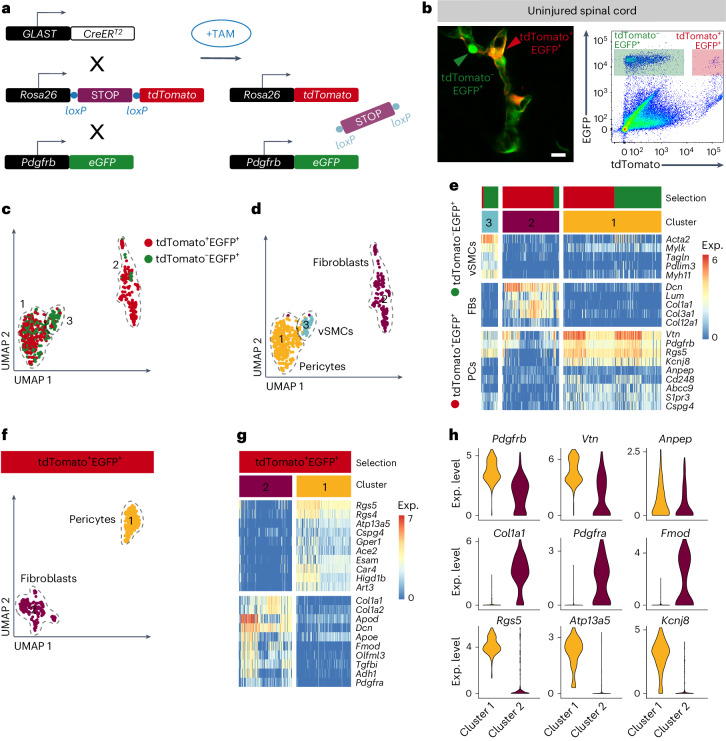
GLAST-expressing perivascular cells of the uninjured spinal cord comprise both pericytes and fibroblasts. **a**, GLAST-expressing perivascular cells can be labeled by tamoxifen-mediated genetic recombination in adult *GLAST-CreER*^*T2*^ transgenic mice carrying *R26R-tdTomato* reporter alleles, in combination with the *Pdgfrb-eGFP* reporter line. **b**, Left, both tdTomato^+^EGFP^+^ and tdTomato^−^EGFP^+^ cells are associated with blood vessels in the uninjured spinal cord of adult *GLAST-CreER*^*T2*^;*R26R-tdTomato*;*Pdgfrb-eGFP* mice. Right, thoracic spinal cord segments were dissected out and dissociated into a single-cell suspension, and tdTomato^+^EGFP^+^ and tdTomato^−^EGFP^+^ cells were isolated by fluorescence-activated single-cell sorting (FACS) for downstream single-cell RNA sequencing; the FACS plot is representative of four independent experiments. **c**, UMAP plot for dimension reduction of spinal cord single-cell data color-coded for 205 tdTomato^−^EGFP^+^ (green) and 315 tdTomato^+^EGFP^+^ (red) cells. **d**, The same UMAP plot as in **c** but color-coded for Seurat clustering of single cells into three distinct clusters. **e**, Heat map of single-cell RNA-sequencing gene expression data of perivascular cells from the uninjured spinal cord separated according to tdTomato^−^EGFP^+^ (green) and tdTomato^+^EGFP^+^ (red) origin of cells (selection) and clusters 1–3. All tdTomato^+^EGFP^+^ cells express established perivascular cell markers, such as vitronectin (*Vtn*), *Pdgfrb* and CD13 (*Anpep*), whereas *Rgs5* is highly expressed in cluster 1 and lowly expressed in cluster 2. Other pericyte markers, such as *Kcnj8*, *Abcc9* and *Cd248*, are mostly expressed in cluster 1. Fibroblast markers are largely expressed in cluster 2, and vSMC markers are mostly expressed in cluster 3. **f**, UMAP plot for dimension reduction of tdTomato^+^EGFP^+^ cells color-coded for cells of clusters 1 and 2. **g**, Heat map of differentially expressed genes between tdTomato^+^EGFP^+^ cells from clusters 1 and 2. **h**, Violin plots showing the expression levels of selected perivascular marker genes with similar or differential expression between tdTomato^+^EGFP^+^ cells in clusters 1 and 2. TAM, tamoxifen; PCs, pericytes; FBs, fibroblasts; vSMCs, vascular smooth muscle cells; Exp., expression. Scale bar: 10 μm (**b**).

Lineage tracing of GLAST-expressing perivascular cells using *GLAST-CreER*^*T2*^ transgenic mice demonstrates up to 95% contribution to scar-forming fibroblasts after a complete spinal crush, a model of nonpenetrating SCI^10^. To define the molecular identity of GLAST-expressing perivascular cells, we crossed *GLAST-CreER*^*T2*^ mice to *R26R-tdTomato* Cre reporter mice and the *Pdgfrb-eGFP* reporter line, which drives enhanced GFP (EGFP) expression in all PDGFRβ^+^ perivascular cells (pericytes, vSMCs and fibroblasts). We then isolated equal numbers of GLAST^+^ (tdTomato^+^EGFP^+^) and GLAST^−^ (tdTomato^−^EGFP^+^) perivascular cells from uninjured spinal cords (Fig. 1a,b and Supplementary Fig. 1a). Sorted cells were subjected to high-sensitivity single-cell gene expression profiling using Smart-seq^16–18^, in which 520 cells passed the downstream quality controls and were further analyzed.

To classify the sorted populations, we used marker profiles for pericytes, fibroblasts, vSMCs, astrocytes, oligodendrocytes and oligodendrocyte progenitor cells^19^ and compared marker gene expression between clusters. Cells in cluster 1 consisted of similar numbers of tdTomato^+^EGFP^+^ and tdTomato^−^EGFP^+^ cells and were identified as pericytes based on high expression of pericyte marker genes and low expression of fibroblast and vSMC markers (Fig. 1c–e). Astrocyte and oligodendroglia lineage markers were absent in cluster 1 (Supplementary Fig. 1b). Cluster 2 contained mainly tdTomato^+^EGFP^+^ cells, which were classified as perivascular fibroblasts (Fig. 1c–e) based on lower expression of the pericyte marker genes *Cspg4*, *S1pr3*, *Cd248*, *Kcnj8*, *Abcc9* and *Rgs5* and higher expression of the fibroblast markers *Col1a1*, *Col3a1*, *Lum*, *Dcn* and *Pdgfra* compared to cells in cluster 1 (Fig. 1e and Supplementary Fig. 1a). PDGFRα is also expressed by oligodendrocyte progenitor cells^20^, but cluster 2 cells did not express other oligodendroglia lineage marker genes (Supplementary Fig. 1a). We also detected the expression of the astrocyte marker genes *Gja1* and *Gjb6*, encoding the gap junction proteins connexin 43 and connexin 30, respectively^21,22^, but no additional astrocyte marker genes were detected to be expressed (Supplementary Fig. 1a). Furthermore, we validated *Col1a1*, *Pdgfra* and *Gjb6* expression in *Pdgfrb*^+^ perivascular cells (Supplementary Fig. 1c–e), in line with previous reports^23–25^. Cluster 3 contained mostly tdTomato^−^EGFP^+^ cells that were classified as vSMCs based on the high expression of *Pdlim*, *Myh11*, *Mylk*, *Acta2* (encoding α-smooth muscle actin (αSMA)) and *Tagln* (encoding smooth muscle protein 22-α (SM22α)) (Fig. 1c–e). Taking these findings together, we found that most GLAST-expressing perivascular cells are transcriptionally classified as pericytes and that current clustering methods do not distinguish them from GLAST^−^ pericytes (Fig. 1c), in line with a previous report^11^. Notably, we found a subset of GLAST^+^ cells clustering separately from pericytes and presenting a fibroblast-like gene expression profile, revealing heterogeneity within GLAST-expressing perivascular cells. GLAST^+^ cells were distinct from vSMCs (Fig. 1d,e). The cell-type classification was confirmed by integrating the gene expression profiles of all tdTomato^+^EGFP^+^ cells with a published single-cell dataset^11^ that molecularly defined mural cells and fibroblasts from the adult mouse brain (Supplementary Fig. 2a–c). Accordingly, 16% of the cells classified as pericytes by Vanlandewijck et al.^11^ showed expression of GLAST (*Slc1a3*) (Supplementary Fig. 3a). *Slc1a3*-expressing pericytes showed a similar topology to *Spp1-*, *Itih5-* and *Apod-*expressing cells, whereas *Pdgfa* and *Casq2* expression reflected the *Slc1a3*^−^ topology. These results indicate a gene expression gradient with vSMCs on one side and *Slc1a3*^+^ or *Spp1*^+^ pericytes on the other (Supplementary Fig. 3a). Consistent with this, we detected neuron–glial antigen 2 (NG2; encoded by *Cspg4*) expression but not *Spp1* expression in tdTomato^+^EGFP^+^ capillary pericytes. Nonetheless, tdTomato^+^EGFP^+^ cells of the arteriole–capillary transitional zone with projections into the capillary bed expressed *Spp1* (Supplementary Fig. 3b,c).

Reclustering of tdTomato^+^EGFP^+^ cells alone confirmed that GLAST^+^ cells encompass pericytes and fibroblasts (Fig. 1f,g). Gene Ontology (GO) enrichment analysis revealed gene expression related to vasculogenesis, transport across and maintenance of the blood–brain barrier, regulation of local adhesion assembly and regulation of phosphatidylinositol 3-kinase signaling in GLAST^+^ pericytes; in contrast, fibroblasts showed higher expression of genes related to ECM organization, regulation of insulin-like growth factor receptor signaling pathway, cell migration and fibroblast proliferation (Supplementary Fig. 4).

We identified fibroblast-enriched expression of *Col1a1*, *Pdgfra* and *Fmod*, among others (Fig. 1g,h). Owing to the absence of expression in other cell types of the adult CNS, we selected *Col1a1* as a fibroblast-specific marker for downstream cell-specific targeting strategies.

### Targeting of *Col1a1*-expressing perivascular fibroblasts

**Fig. 2:**
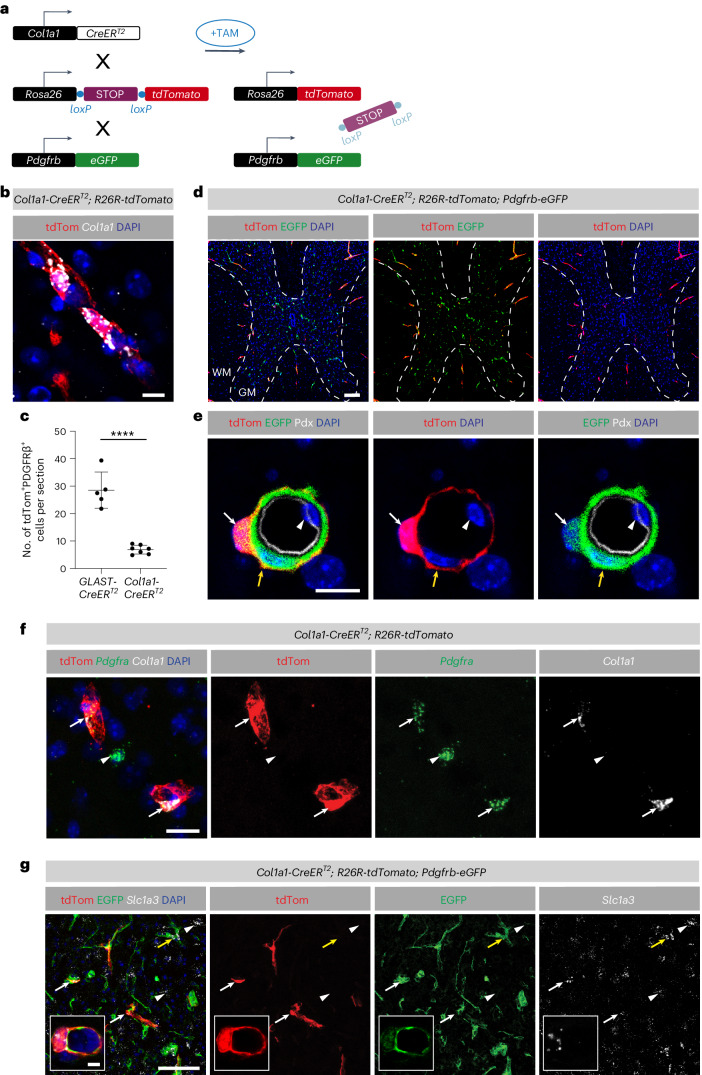
Inducible *Col1a1-CreER*^*T2*^ transgenic mice target perivascular fibroblasts in the uninjured mouse spinal cord. **a**, *Col1a1*^+^ perivascular cells can be labeled by tamoxifen-mediated genetic recombination in adult *Col1a1*-*CreER*^*T2*^ transgenic mice carrying *R26R-tdTomato* reporter alleles, in combination with the *Pdgfrb-eGFP* reporter line. **b**, Detection of *Col1a1* mRNA in tdTomato^+^ cells in the uninjured spinal cord of *Col1a1-CreER*^*T2*^;*R26R-tdTomato* mice. **c**, Quantification of the average number of tdTomato^+^ cells per transverse spinal cord section (20-μm-thick) in uninjured *Col1a1-CreER*^*T2*^;*R26R-tdTomato* (mean ± s.d., 6.9 ± 1.6) and *GLAST-CreER*^*T2*^;*R26R-tdTomato* (mean ± s.d., 28.5 ± 6.6) mice. **d**,**e**, In the uninjured spinal cord, *Col1a1-CreER*^*T2*^ tdTomato^+^ cells express *Pdgfrb-*EGFP (white arrow) (**d**) and surround the blood vessel wall (marked with podocalyxin, white arrowhead) together with *Pdgfrb*-EGFP^+^ mural cells (yellow arrow) (**e**). **f**, *Col1a1-CreER*^*T2*^ tdTomato^+^ cells coexpress *Col1a1* and the fibroblast marker *Pdgfra* (white arrows). The *Pdgfra* expression level in tdTomato^+^*Col1a1*^+^ fibroblasts is lower than in neighboring tdTomato^−^*Col1a1*^−^;*Pdgfra*^+^ cells (white arrowhead), which are presumably oligodendrocyte precursor cells. **g**, *Slc1a3* (GLAST) is expressed by tdTomato^+^EGFP^+^ perivascular fibroblasts (white arrows) in the spinal cord of *Col1a1-CreER*^*T2*^;*R26R-tdTomato*;*Pdgfrb-eGFP* mice. In addition, *Slc1a3* expression is detected in tdTomato^−^EGFP^−^ cells (white arrowheads), which are mostly astrocytes, as well as in tdTomato^−^EGFP^+^ perivascular cells (yellow arrows). The insets show the close-up view of a tdTomato^+^EGFP^+^*Slc1a3*^+^ perivascular fibroblast. Pdx, podocalyxin; WM, white matter; GM, gray matter; tdTom, tdTomato. Scale bars: 100 μm (**d**), 20 μm (**f**, **g**), 10 μm (**e**, **b**) and 5 μm (insets in **g**). Data are shown as mean ± s.d. *n* = 5 (*GLAST-CreER*^*T2*^) and *n* = 7 (*Col1a1-CreER*^*T2*^) animals in **c**. ****P < 0.0001 by two-sided, unpaired Student’s *t* test in **c**. Dashed lines in **d** outline the spinal cord gray matter. Cell nuclei are labeled with 4′,6′-diamidino-2-phenylindole dihydrochloride (DAPI). Images are representative of two independent experiments. All images show transverse sections. Source data are provided as a source data file. Source data

To specifically target *Col1a1*-expressing fibroblasts in the adult CNS, we used an inducible *Col1a1-CreER*^*T2*^ mouse line (Methods) and crossed it to the *R26R-tdTomato* reporter mouse line. In some cases, this line was further crossed to the *Pdgfrb-eGFP* reporter mouse line (Fig. 2a). Recombination of *Col1a1-CreER*^*T2*^;*R26R-tdTomato* mice led to faithful expression of tdTomato in *Col1a1*^+^ cells, as confirmed by RNAscope in situ hybridization (Fig. 2b). The average recombination efficacy was 71.2 ± 5.7% and 82.8 ± 3.7% (mean ± s.d.) in the gray and white matter, respectively (Supplementary Fig. 5a–d). The *Col1a1-CreER*^*T2*^ line recombined 6.9 ± 1.6 (mean ± s.d.) perivascular fibroblasts per section, whereas the *GLAST-CreER*^*T2*^ line, which targets GLAST^+^ pericytes and fibroblasts, recombined 28.5 ± 6.6 (mean ± s.d.) cells (Fig. 2c). *Col1a1-CreER*^*T2*^ tdTomato^+^ cells coexpressed *Pdgfrb*-EGFP and *Pdgfra* (Fig. 2d–f). We found that 98.3 ± 2.9% (mean ± s.d.) of the *Col1a1-CreER*^*T2*^ tdTomato^+^ fibroblasts expressed *Slc1a3* (GLAST) in the uninjured spinal cord (Fig. 2g and Supplementary Fig. 5e,f). We also detected the expression of *Gjb6* in tdTomato^+^ perivascular fibroblasts, validating the scRNA-sequencing results (Supplementary Fig. 6a). Within the population of GLAST-expressing cells (tdTomato^+^), *Col1a1* expression was highest in perivascular cells on large-caliber penetrating blood vessels and significantly lower on smaller-caliber blood vessels. *GLAST-CreER*^*T2*^ tdTomato^+^ cells associated with the microvasculature had no detectable *Col1a1* expression (Supplementary Fig. 6b–d). Together, these results indicate that *Col1a1* and GLAST are coexpressed in perivascular fibroblasts and can be genetically labeled with the *Col1a1-CreER*^*T2*^ and *GLAST-CreER*^*T2*^ transgenic mouse lines, whereas capillary-associated pericytes do not express *Col1a1* and are captured only in *GLAST-CreER*^*T2*^ mice.

### Anatomical distribution of fibroblasts and GLAST^+^ pericytes

**Fig. 3:**
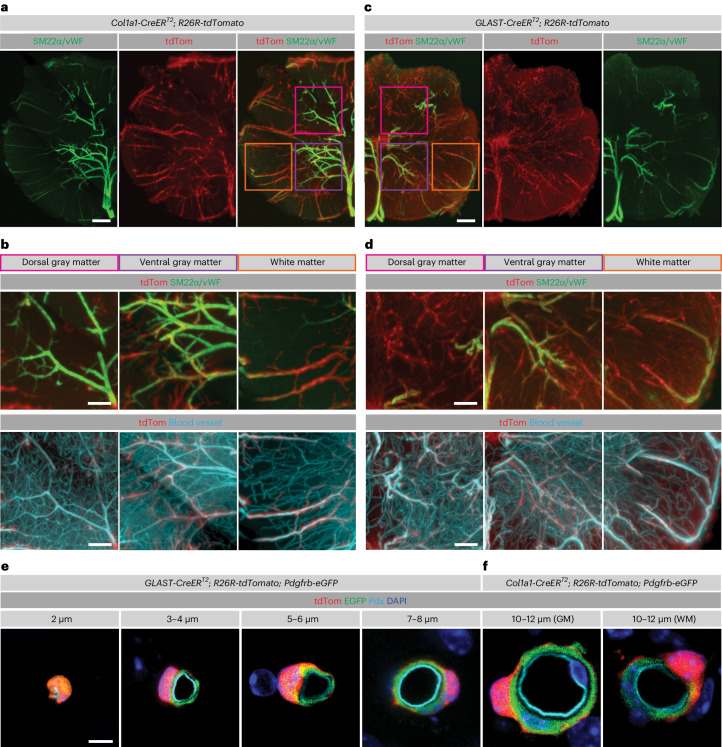
Distribution of perivascular fibroblasts and GLAST^+^ pericytes in the uninjured mouse spinal cord. **a**,**c**, Distribution of *Col1a1*-*CreER*^*T2*^ tdTomato^+^ cells (**a**) and *GLAST-CreER*^*T2*^ tdTomato^+^ cells (**c**) revealed by volumetric imaging of 500-μm-thick uninjured spinal cord thoracic segments costained with antibodies against SM22α and vWF for labeling of arterioles/arteries and venules/veins, respectively. **b**,**d**, Enlarged images of the spinal cord dorsal gray matter, ventral gray matter and white matter (from the boxed regions in **a** and **c**) showing that *Col1a1-CreER*^*T2*^ tdTomato^+^ cells (**b**) do not extend as far along arteriolar branches as *GLAST-CreER*^*T2*^ tdTomato^+^ cells (**d**). The blood vessel lumen was labeled by transcardial perfusion with Alexa Fluor 647-conjugated BSA in a gelatin solution. **e**, *GLAST-CreER*^*T2*^ tdTomato^+^ cells expressing *Pdgfrb*-EGFP can be found in close contact with endothelial cells on capillaries and small microvessels, on blood vessels of intermediate diameter and surrounding tdTomato^−^EGFP^+^ mural cells on larger blood vessels. **f**, *Col1a1-CreER*^*T2*^ tdTomato^+^ fibroblasts express *Pdgfrb*-EGFP and are preferentially located along large-diameter blood vessels, surrounding tdTomato^−^EGFP^+^ mural cells in the spinal cord gray matter (GM) and white matter (WM). The inner diameter of blood vessels in **e** and **f** was measured in formaldehyde-fixed, cryopreserved tissue sections. Scale bars: 200 μm (**a**, **c**), 100 μm (**b**, **d**) and 5 μm (**e**, **f**). Cell nuclei are labeled with DAPI. Images are representative of three independent experiments. All images show transverse sections.

**Extended Data Fig. 1:**
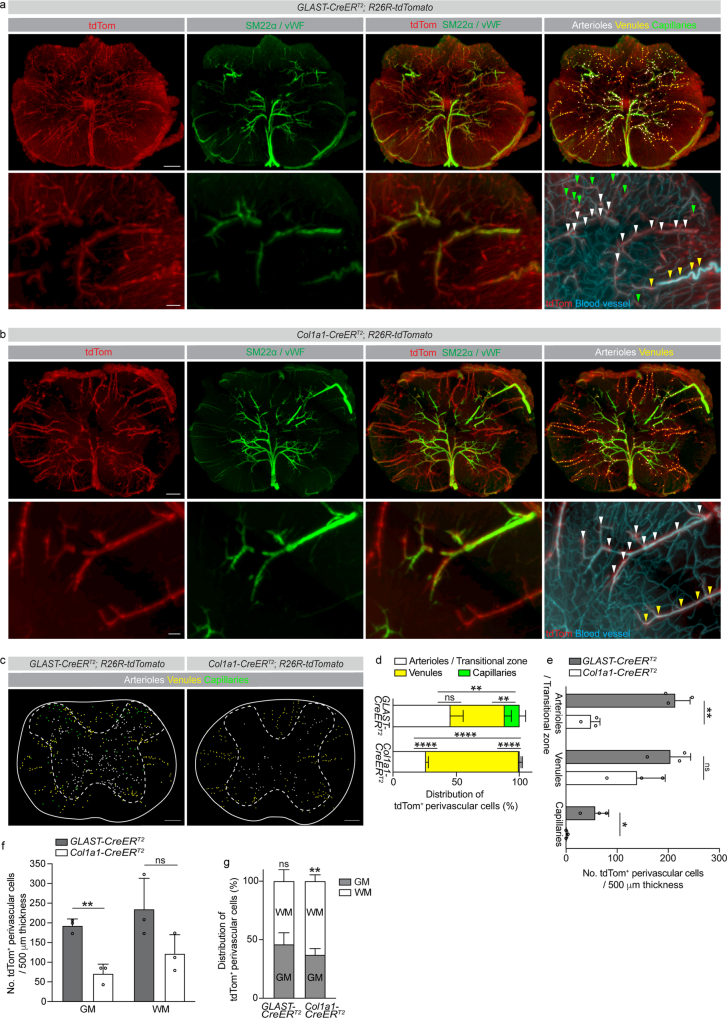
Perivascular fibroblasts and GLAST^−^expressing pericytes distribute differently along the vascular tree and between grey matter and white matter in the uninjured spinal cord. Distribution of GLAST-CreER^T2^ tdTomato^+^ (**a**) and Col1a1-CreER^T2^ tdTomato^+^ (**b**) cells along arterioles (white arrowheads/dots), venules (yellow arrowheads/dots) and capillaries (green arrowheads/dots) revealed by volumetric imaging of 500 μm thick uninjured spinal cord thoracic segments co-stained with antibodies against SM22α (arterioles/arteries) and vWF (large ascending venules/veins) (upper panel) and 200 μm thick enlarged image of the dorsal grey matter (lower panel). The blood vessel lumen was labeled by transcardial perfusion with Alexa Fluor 647-conjugated bovine serum albumin in a gelatin solution. (**c**) Transverse sections of the spinal cord (same sections as shown in (**a**) and (**b**)) showing the location of GLAST-CreER^T2^ tdTomato^+^ cells and Col1a1-CreER^T2^ tdTomato^+^ cells within a 500 μm thick uninjured spinal cord thoracic segment (each dot represents a cell, color-coded for the vessel type the cell resides on). Solid lines outline the spinal cord parenchyma, dashed lines outline the grey matter. (**d**) Distribution of tdTomato^+^ perivascular cells along arterioles (including the arteriole-capillary transitional zone), venules and capillaries, represented as a percentage of total tdTomato^+^ perivascular cells. (**e**) Quantification of the number of tdTomato^+^ perivascular cells per 500 μm thick uninjured spinal cord thoracic segments. (**f**) Quantification of the number of tdTomato^+^ perivascular cells within the grey and white matter per 500 μm thick uninjured spinal cord thoracic segments. (**g**) Distribution of tdTomato^+^ perivascular cells in the spinal cord grey and white matter, represented as a percentage of total tdTomato^+^ perivascular cells. There are significantly more Col1a1-CreER^T2^ tdTomato^+^ cells in the white matter than in the grey matter. Scale bars: 200 μm (a and b upper panel, c) and 50 μm (a and b lower panel). Data shown as mean + SD. n = 3 (GLAST-CreER^T2^), n = 3 (Col1a1-CreER^T2^) animals in (**d**-**g**). ns=0.688, **p = 0.006 (arterioles-capillaries), **p = 0.0017 (venules-capillaries), ****p < 0.0001 (**d**), **p = 0.001, ns=0.1705, *p = 0.0214 (**e**), **p = 0.0021, ns=0.1008 (**f**), ns=0.3832, **p = 0.004 (**g**) by two^−^sided, unpaired Student’s t test. All images show transverse sections. Images are representative of three independent experiments. Source data are provided as a Source Data file. Source data

To determine the distribution of *Col1a1*^+^ and GLAST^+^ cells along the spinal cord vasculature, we used optical tissue clearing and imaged the thoracic spinal cord segments of recombined *GLAST-CreER*^*T2*^;*R26R-tdTomato* and *Col1a1-CreER*^*T2*^;*R26R-tdTomato* mice using light-sheet microscopy. The blood vessel lumen was labeled by transcardial perfusion with Alexa Fluor 647-conjugated BSA. Arteries and arterioles were marked with an antibody against SM22α, and veins and venules were marked with an antibody against von Willebrand factor (vWF) (Fig. 3a–d, Extended Data Fig. 1a,b and Supplementary Fig. 7a,b). We found 26.1 ± 1.8% (mean ± s.d.) *Col1a1-CreER*^*T2*^ tdTomato^+^ cells on higher-order penetrating arterioles and arteriolar branches and 73.3 ± 2.7% (mean ± s.d.) on venules, whereas they were absent from the capillary network (Fig. 3a,b,f and Extended Data Fig. 1b–e). *GLAST-CreER*^*T2*^ tdTomato^+^ cells were found in the same positions along arterioles and venules (Fig. 3c,d), confirming the overlap of GLAST and *Col1a1* expression in fibroblasts (Figs. 1c–h and 2f,g and Supplementary Fig. 6b–d). In addition, the *GLAST-CreER*^*T2*^ line targeted perivascular cells on smaller arteriolar branches and the capillary bed (Fig. 3c–e and Extended Data Fig. 1a,c–e). Overall, 42.7 ± 5.3% (mean ± s.d.) of *GLAST-CreER*^*T2*^ tdTomato^+^ cells were associated with venules, 45.5 ± 9.9% (mean ± s.d.) were distributed along the arteriolar tree including the postarteriolar transitional zone and 11.8 ± 4.7% (mean ± s.d.) were associated with capillaries (Fig. 3c–e and Extended Data Fig. 1a,c–e). Gray matter regions of the spinal cord contain most arteriolar branches and a higher density of capillaries than white matter regions, whereas penetrating arterioles and venules mostly run in the white matter. These anatomical characteristics result in a higher number of *GLAST-CreER*^*T2*^ tdTomato^+^ cells than *Col1a1-CreER*^*T2*^ tdTomato^+^ cells in gray matter regions (Fig. 3a–d and Extended Data Fig. 1f,g). Both lines showed a higher number of tdTomato^+^ cells in the ventral than in the dorsal side of the spinal cord (Fig. 3a,c).

### Ultrastructural features of fibroblasts and GLAST^+^ pericytes

**Fig. 4:**
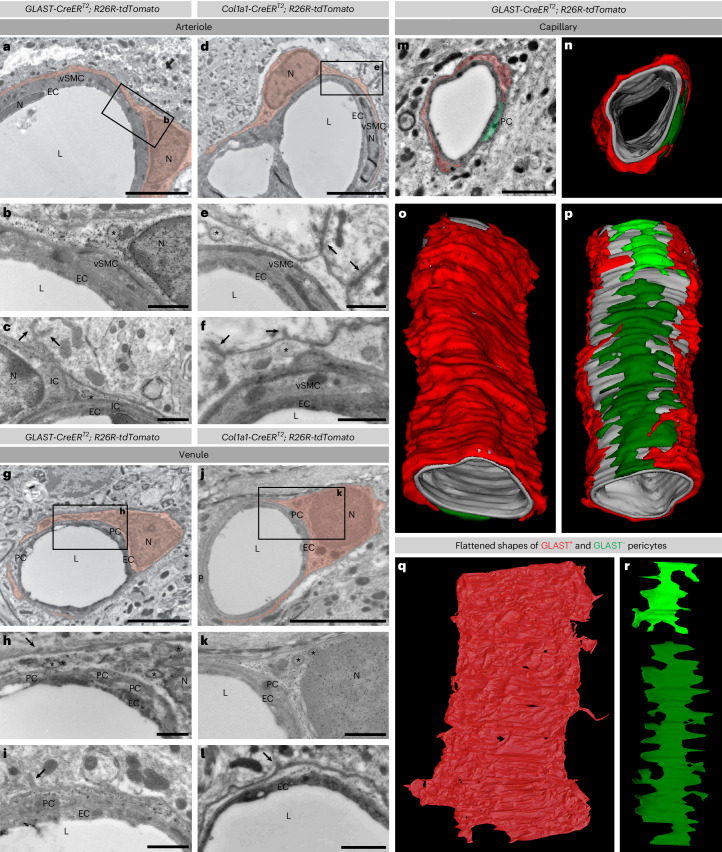
Electron microscopic comparison of GLAST- and *Col1a1*-expressing perivascular cells in the uninjured mouse spinal cord. **a**–**l**, Immunogold electron microscopy images showing GLAST-expressing (**a**–**c**, **g**–**i**) and *Col1a1-*expressing (**d**–**f**, **j**–**l**) cells on two different arterioles (**a**–**f**) and venules (**g**–**l**). Immunogold particles label *GLAST-CreER*^*T2*^ tdTomato^+^ and *Col1a1-CreER*^*T2*^ tdTomato^+^ perivascular cells (pseudocolored red). **a**,**b**,**d**,**e**, Both GLAST- and *Col1a1*-expressing cells have an oval nucleus and thin processes surrounding the vascular wall, including vSMCs. GLAST-expressing (**a**–**c**) and *Col1a1*-expressing (**d**–**f**) cells show similar structural features, including basal lamina protrusions (arrows), bright cytoplasm and coated vesicles (asterisk). High-magnification micrographs of the insets in **a** and **d** are shown in **b** and **e**, respectively. **c**,**f**, A portion of a GLAST-expressing cell (**c**) and a *Col1a1*-expressing cell (**f**) in two other arterioles. The same morphological features are observed in GLAST- and *Col1a1-*expressing cells on venules: oval to round nucleus, thin processes surrounding the vessel, bright cytoplasm (**g**, **j**); basal lamina protrusions (arrows) and coated vesicles (asterisk) (**h**, **i**, **k**, **l**). **i**,**l**, Specific for the venules are areas where GLAST- and *Col1a1-*expressing cells are directly apposed to endothelial cells with only the basal membrane separating them. **m**–**r**, 3D reconstruction of a GLAST-expressing cell on a capillary (tdTomato^+^ cell; pseudocolored red) in comparison to two pericytes (pseudocolored green). **m**,**n**, The 3D model spans a length of 13.2 µm of the capillary. **o**,**p**, Both the GLAST-expressing cell and the GLAST^−^ pericytes are embedded in the vascular wall directly apposing the endothelial tube, occupying a mutually exclusive space with little overlap. At places where they overlap, the GLAST-expressing cell is always abluminal. **q**,**r**, Unwrapped GLAST-expressing and GLAST^−^ pericytes to visualize process morphology. GLAST-expressing cells (**q**) have a distinctly wide appearance occupying a larger surface of the blood vessel wall and fewer, less well-defined finger-like extensions compared to neighboring GLAST^−^ pericytes (**r**). N, nucleus; L, lumen; EC, endothelial cell; PC, GLAST^−^ pericyte; IC, pericyte–vSMC intermediary cell; asterisk, coated vesicle; double arrow, basal lamina protrusion. Scale bars: 5 µm (**a**, **d**, **g**, **j**) and 1 µm (**b**, **c**, **e**, **f**, **h**, **i**, **k**, **l**, **m**). Images are representative of two independent experiments. All electron microscopy images show transverse sections.

We used immunogold labeling to identify *GLAST-CreER*^*T2*^ tdTomato^+^ and *Col1a1-CreER*^*T2*^ tdTomato^+^ cells in the uninjured spinal cord at the ultrastructural level. Along large arterioles, both lines labeled cells embedded in the outermost layer of the basal lamina, surrounding vSMCs, with a light cytoplasm, an oval-shaped nucleus, large light vesicular organelles and basal lamina protrusions extending into astrocytic endfeet (Fig. 4a–f), classifying them as perivascular fibroblasts^26^. On the venular side, immunogold-labeled tdTomato^+^ cells from both transgenic lines adhered to the vascular basal lamina and presented a light cytoplasm and membrane protrusions toward the abluminal side. Frequently, we observed labeled cells directly juxtaposed to endothelial cells, only separated by the basement membrane (Fig. 4g–l). In addition, the *GLAST-CreER*^*T2*^ transgenic line labeled perivascular cells in the transitional zone between smaller arteriole branches and the capillary bed, as well as in the capillary bed (Fig. 3e). These cells were embedded within the vascular basal lamina, juxtaposed to endothelial cells (Fig. 4m)^8,10^. At positions where processes intersected, *GLAST-CreER*^*T2*^ tdTomato^+^ cells were commonly located abluminal to other, nonrecombined pericytes (Fig. 3e)^8^. When transitioning to the capillary bed, *GLAST-CreER*^*T2*^ tdTomato^+^ cells no longer intersected with other pericytes. A three-dimensional (3D) serial reconstruction of 13.2-µm capillary length revealed that *GLAST-CreER*^*T2*^ tdTomato^+^ cells were directly abutting the endothelial tube and shared the endothelial basement membrane, like neighboring nonrecombined pericytes (Fig. 4m–p). Interestingly, while nonrecombined pericytes presented a classic morphology with finger-shaped processes extending radially^27^, *GLAST-CreER*^*T2*^ tdTomato^+^ cells covered the endothelial surface more homogeneously (Fig. 4o–r). Moreover, the cytoplasm of *GLAST-CreER*^*T2*^ tdTomato^+^ pericytes was less electron dense compared to neighboring pericytes (Fig. 4m). In summary, both *GLAST-CreER*^*T2*^ and *Col1a1-CreER*^*T2*^ transgenic lines target perivascular fibroblasts with similar ultrastructural features and perivascular position on larger arterioles and venules. In addition, the *GLAST-CreER*^*T2*^ line targets a population of GLAST^+^*Col1a1*^−^ cells, which are embedded in the vascular basement membrane along capillaries and the arteriole–capillary transitional zone. Based on their gene expression profile (Fig. 1e) and location within the vascular basal lamina juxtaposed to the endothelial cells (Fig. 4m–p), these cells can be classified as pericytes^14^.

### Region-dependent contribution to fibrotic scarring

**Fig. 5:**
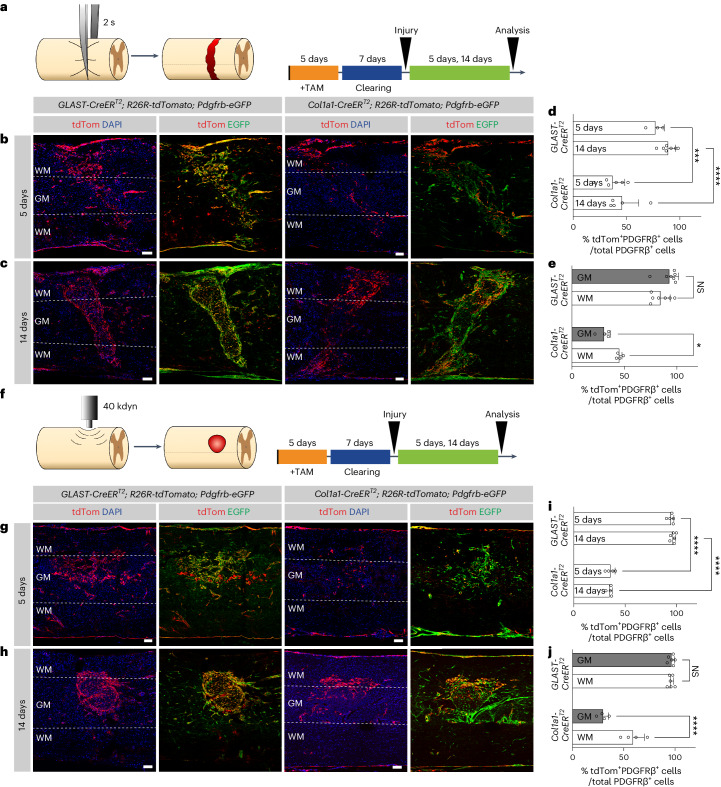
Perivascular fibroblasts contribute more extensively to white matter scarring, whereas GLAST-expressing pericytes generate fibrotic scar tissue in the gray matter after SCI. **a**, SCI lesion model (complete spinal crush at thoracic level 10 (T10)) and experimental timeline used for lineage tracing of GLAST- and *Col1a1*-expressing perivascular cells. **b**,**c**, Distribution of tdTomato^+^EGFP^+^ and tdTomato^−^EGFP^+^ cells in *GLAST-CreER*^*T2*^ and *Col1a1-CreER*^*T2*^;*R26R-tdTomato*;*Pdgfrb-eGFP* mice at 5 days (**b**) and 14 days (**c**) after the complete spinal crush. The *Pdgfrb-eGFP* reporter line drives EGFP expression in PDGFRβ-expressing perivascular and stromal cells. **d**, Percentage of PDGFRβ^+^ cells that express tdTomato out of the total PDGFRβ^+^ stromal cells in the lesion core at 5 and 14 days after injury. **e**, Percentage of tdTomato^+^PDGFRβ^+^ cells out of the total PDGFRβ^+^ stromal cells in gray and white matter regions of the spinal cord at 14 days after injury. **f**, Lesion model (mild contusion at T9) and experimental timeline used for lineage tracing of GLAST- and *Col1a1*-expressing perivascular cells. **g**,**h**, Distribution of tdTomato^+^EGFP^+^ and tdTomato^−^EGFP^+^ cells in *GLAST-CreER*^*T2*^ and *Col1a1-CreER*^*T2*^;*R26R-tdTomato*;*Pdgfrb-eGFP* mice at 5 days (**g**) and 14 days (**h**) after the mild spinal cord contusion. **i**, Percentage of PDGFRβ^+^ cells that express tdTomato out of the total PDGFRβ^+^ stromal cells in the lesion core at 5 and 14 days after injury. **j**, Percentage of tdTomato^+^PDGFRβ^+^ cells out of the total PDGFRβ^+^ stromal cells in gray and white matter regions of the spinal cord at 14 days after injury. All scale bars: 100 µm. Data are shown as mean ± s.d. Crush: *GLAST-CreER*^*T2*^ *n* = 3 (5 days) and *n* = 7 (14 days), *Col1a1-CreER*^*T2*^ *n* = 6 (5 days) and *n* = 5 (14 days) animals in **d** and *n* = 7 (*GLAST-CreER*^*T2*^ 14 days) and *n* = 4 (*Col1a1-CreER*^*T2*^ 14 days) animals in **e**. Contusion: *GLAST-CreER*^*T2*^ *n* = 5 (5 days) and *n* = 6 (14 days), *Col1a1-CreER*^*T2*^ *n* = 4 (5 days) and *n* = 4 (14 days) animals in **i** and *n* = 6 (*GLAST-CreER*^*T2*^ 14 days) and *n* = 4 (*Col1a1-CreER*^*T2*^ 14 days) animals in **j**. ***P = 0.0002, ****P < 0.0001 (**d**), *P = 0.0356, NS (no significance) = 0.1096 (**e**), ****P < 0.0001 (**i**), NS = 0.9804, ****P < 0.0001 (**j**) by one-way analysis of variance (ANOVA) followed by Šídák’s multiple-comparisons test. Dashed lines in **b**, **c**, **g** and **h** outline the gray matter–white matter border. Cell nuclei are labeled with DAPI. Images are representative of two independent experiments. All images show sagittal sections. Source data are provided as a source data file. Source data

To investigate the contribution of GLAST^+^ pericytes and perivascular fibroblasts to scar-forming fibroblasts in the context of SCI, we recombined *GLAST-CreER*^*T2*^;*R26R-tdTomato*;*Pdgfrb-eGFP* and *Col1a1-CreER*^*T2*^;*R26R-tdTomato*;*Pdgfrb-eGFP* mice with tamoxifen, followed by a clearing period to ensure specificity^3,28^, and generated complete spinal crush lesions at a low thoracic level (Fig. 5a). GLAST-expressing pericytes and fibroblasts contributed 77.1 ± 8.2% and 89.7 ± 7.0% (mean ± s.d.) to all PDGFRβ^+^ scar-forming fibroblasts at 5 and 14 days after SCI, respectively (Fig. 5b–d), as observed previously^10^. In comparison, perivascular fibroblasts, targeted by the *Col1a1-CreER*^*T2*^ transgenic line, contributed to 37.0 ± 11.3% and 45.9 ± 15.6% (mean ± s.d.) of all PDGFRβ^+^ scar-forming fibroblasts at 5 and 14 days after SCI, respectively (Fig. 5b–d). These results indicate that, although virtually all scar-forming fibroblasts upregulate *Col1a1* after SCI (Supplementary Fig. 8a–d), only a fraction of these cells are derived from *Col1a1*-expressing perivascular fibroblasts.

While *GLAST-CreER*^*T2*^ tdTomato^+^EGFP^+^ cells were distributed homogeneously throughout the fibrotic scar tissue, we observed that *Col1a1-CreER*^*T2*^ tdTomato^+^EGFP^+^ cells contributed significantly less to scar tissue in gray matter regions compared to white matter regions (Fig. 5b,c,e and Supplementary Fig. 8c,d). These observations suggest that perivascular fibroblasts, which are primarily located along large blood vessels in the spinal cord white matter, react locally to injury. In turn, GLAST^+^ pericytes, which are predominantly located in the spinal cord gray matter, contribute more extensively to gray matter scarring.

To validate the regional differences in scar contribution between GLAST^+^ pericytes and perivascular fibroblasts, we generated mild contusions (40 kdyn) at a low thoracic level, which caused fibrotic scar tissue formation in the central gray matter and immediately adjacent white matter regions (Fig. 5f–h). *GLAST-CreER*^*T2*^ tdTomato^+^ cells contributed to 94.7 ± 2.8% and 96.8 ± 2.4% (mean ± s.d.) of all PDGFRβ^+^ stromal fibroblasts at 5 and 14 days after contusion lesions, respectively (Fig. 5g–i). Again, *GLAST-CreER*^*T2*^ tdTomato^+^EGFP^+^ cells showed a similar contribution to fibrotic scarring in gray and white matter regions (Fig. 5g,h,j). In turn, fate-mapped *Col1a1*^+^ fibroblasts contributed to 35.8 ± 4.5% and 35.0 ± 2.5% (mean ± s.d.) of all PDGFRβ^+^ scar-forming fibroblasts at 5 and 14 days after contusion, respectively (Fig. 5g–i). Once more, we observed that the contribution of *Col1a1-CreER*^*T2*^ tdTomato^+^EGFP^+^ cells to fibrotic scarring was more pronounced in white than in gray matter regions (Fig. 5g,h,j).

**Extended Data Fig. 2:**
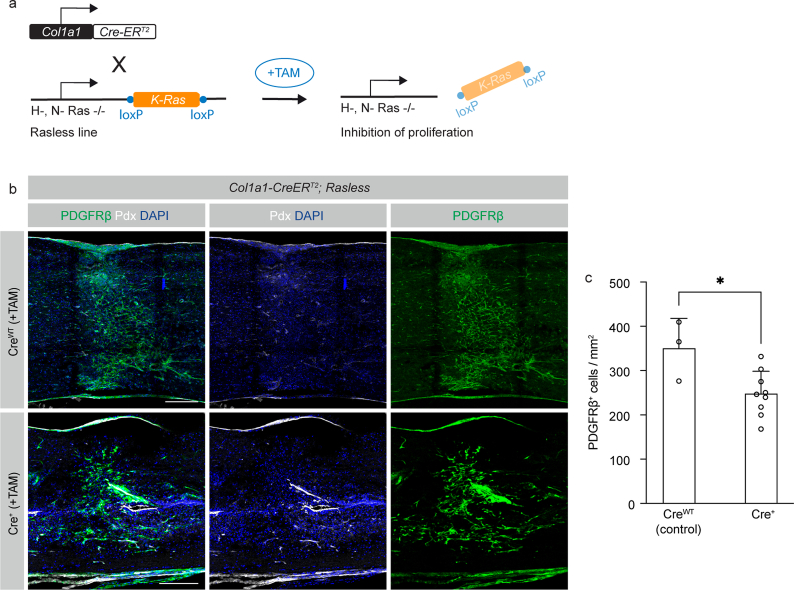
Genetic inhibition of perivascular fibroblast proliferation reduces the total number of PDGFRβ^+^ stromal fibroblasts upon SCI. (**a**) Genetic strategy to inhibit injury-induced proliferation of Col1a1-CreER^T2^ perivascular fibroblasts through cell-specific deletion of floxed K-Ras in mice with H-Ras and N-Ras null alleles (Col1a1-CreER^T2^;Rasless mice) upon tamoxifen-induced genetic recombination. (**b**) Sagittal view of the lesion showing the distribution of PDGFRβ^+^ stromal fibroblasts and blood vessels (podocalyxin^+^, Pdx) in Cre^WT^ and Cre^+^ Col1a1-CreER^T2^;Rasless mice, 7 days after complete spinal cord crush. Both Cre^+^ and Cre^WT^ animals were administered tamoxifen prior to injury, but Cre^WT^ animals did not undergo genetic recombination and served as control. (**c**) The density of scar-forming PDGFRβ^+^ fibroblasts is reduced in Cre^+^ (248.4 ± 50.6 cells/mm2, mean ± SD) compared to Cre^WT^ (350.4 ± 67.9 cells/mm^2^, mean ± SD) Col1a1-Rasless mice. TAM, tamoxifen. Scale bars: 100 µm (**b**). Data shown as mean + SD in (**c**) n = 3 (Cre^WT^), n = 9 (Cre^+^ ) animals. *p = 0.0182 by two-sided, unpaired Student’s t test in (**c**). Cell nuclei are labeled with DAPI. Images are representative of two independent experiments. Source data is provided as a Source Data file. Source data

Next, we asked whether the contribution of perivascular fibroblasts to fibrotic scar tissue can be compensated by GLAST^+^ pericytes. For this, we crossed *Col1a1-CreER*^*T2*^ mice to Rasless mice (*Col1a1*-*CreER*^*T2*^;Rasless), in which injury-induced proliferation of perivascular fibroblasts is inhibited through cell-specific deletion of floxed *K-Ras* in mice with *H-Ras* and *N-Ras* null alleles upon tamoxifen-induced genetic recombination^3,6,10^ (Extended Data Fig. 2a). Both Cre^+^ and Cre^WT^ animals were administered tamoxifen before injury, but Cre^WT^ animals did not undergo genetic recombination and served as controls.

Inhibition of proliferation in Cre^+^ animals reduced the density of scar-forming PDGFRβ^+^ fibroblasts by 29% compared to Cre^WT^ control animals (Extended Data Fig. 2b,c). We did not observe a complete abolishment of scar-forming stromal cell proliferation leading to improper wound healing and the formation of a tissue defect, as reported previously for similar experiments using GLAST-*CreER*^*T2*^;Rasless mice^3,6^. The partial inhibition of fibrotic scarring by rendering *Col1a1*^+^ perivascular fibroblasts unable to proliferate indicates their specific contribution to fibrotic scar tissue.

Together, these results suggest that scar-forming perivascular cells are recruited locally after SCI, with GLAST^+^ pericytes contributing to fibrotic scarring preferentially in gray matter regions and fibroblasts in the white matter of the spinal cord.

### Local recruitment of fibroblasts and pericytes by inflammation

**Fig. 6:**
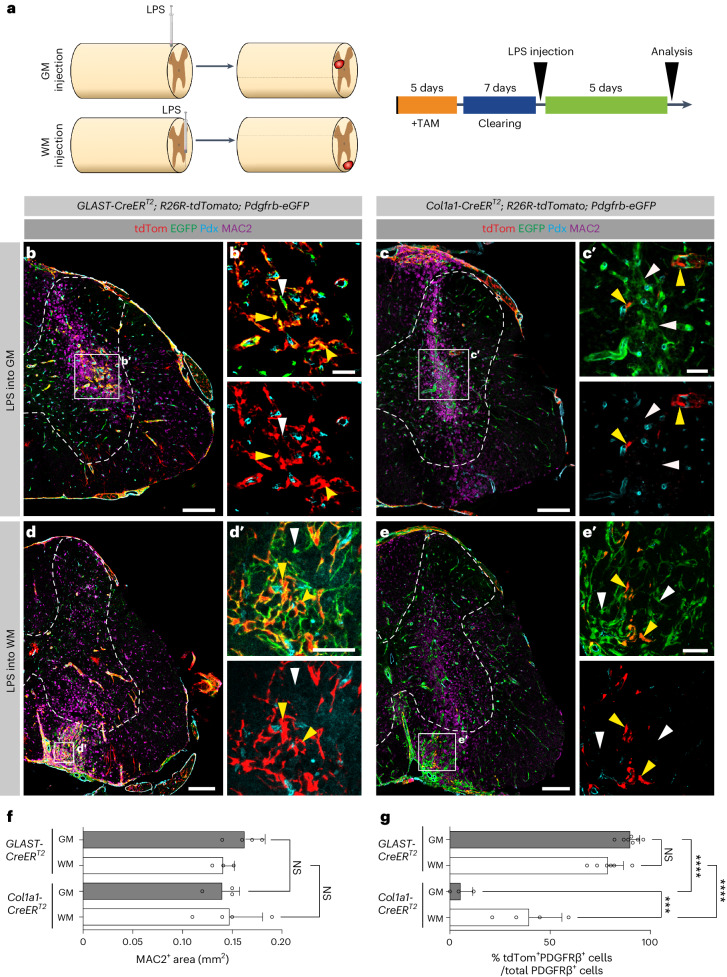
Distinct recruitment of GLAST-expressing pericytes and perivascular fibroblasts in gray and white matter regions of the spinal cord upon focal inflammation. **a**, Schematic outline and experimental timeline used for lineage tracing of GLAST- and *Col1a1*-expressing perivascular cells following LPS injection into the uninjured spinal cord gray matter and white matter. **b**,**c**, Stereotactic injection of LPS into the spinal cord gray matter of *GLAST-CreER*^*T2*^;*R26R-tdTomato*;*Pdgfrb-eGFP* (**b**) and *Col1a1*-*CreER*^*T2*^;*R26R-tdTomato*;*Pdgfrb*-*eGFP* (**c**) mice triggers the recruitment of MAC2^+^ macrophages/microglia and local accumulation of tdTomato^+^EGFP^+^ stromal fibroblasts at 5 days after injection. **b′**, Close-up view of the boxed region in **b** showing that most *Pdgfrb*-EGFP^+^ stromal fibroblasts are tdTomato^+^ (yellow arrowheads), with only a few tdTomato^−^EGFP^+^ cells (white arrowhead) located off blood vessels (podocalyxin^+^). **c′**, Magnified image of the boxed region in **c** showing that almost all *Pdgfrb*-EGFP^+^ stromal fibroblasts are tdTomato^−^ (white arrowheads), indicating that *Col1a1*-expressing perivascular fibroblasts (tdTomato^+^EGFP^+^ cells; yellow arrowheads) do not contribute substantially to gray matter scarring. **d**,**e**, Focal injection of LPS into the spinal cord ventral white matter of *GLAST-CreER*^*T2*^;*R26R-tdTomato*;*Pdgfrb-eGFP* (**d**) and *Col1a1*-*CreER*^*T2*^;*R26R-tdTomato*;*Pdgfrb-eGFP* (**e**) mice leads to recruitment of MAC2^+^ macrophages/microglia and local accumulation of tdTomato^+^EGFP^+^ stromal fibroblasts at 5 days after injection. **d′**,**e′**, Magnified images of the boxed regions in **d** and **e** showing several tdTomato^+^EGFP^+^ stromal fibroblasts (yellow arrowheads) and tdTomato^−^EGFP^+^ stromal cells (white arrowheads) in *GLAST-CreER*^*T2*^;*R26R-tdTomato*;*Pdgfrb-eGFP* (**d′**) and *Col1a1-CreER*^*T2*^;*R26R-tdTomato*;*Pdgfrb-eEGFP* (**e′**) mice. **f**,**g**, Quantification of the inflammatory response by MAC2^+^ cell area (**f**) and percentage of tdTomato^+^PDGFRβ^+^ cells out of the total PDGFRβ^+^ stromal cells (**g**) at 5 days after intraspinal injection of LPS into the gray matter or white matter of *GLAST-CreER*^*T2*^;*R26R-tdTomato*;*Pdgfrb-eGFP* (*GLAST-CreER*^*T2*^) and *Col1a1-CreER*^*T2*^;*R26R-tdTomato*;*Pdgfrb-eGFP* (*Col1a1-CreER*^*T2*^) mice. (**b′**, **c′**, **d′**, **e′**) Upper panel: tdTom, EGFP and Pdx labelling; lower panel: tdTom and Pdx labelling. Scale bars: 500 µm (**b**, **c**, **d**, **e**) and 100 µm (**b′**, **c′**, **d′**, **e′**). Data are shown as mean ± s.d. *Col1a1-CreER*^*T2*^: *n* = 3 (gray matter), *n* = 4 (white matter); *GLAST-CreER*^*T2*^: *n* = 4 (gray matter), *n* = 3 (white matter) animals in **f**. *Col1a1-CreER*^*T2*^: *n* = 3 (gray matter), *n* = 4 (white matter); *GLAST-CreER*^*T2*^: *n* = 7 (gray matter), *n* = 6 (white matter) animals in **g**. NS (gray matter) = 0.5719, NS (white matter) = 0.9788 (**f**) and NS = 0.1624, ***P = 0.007, ****P < 0.0001 (**g**) by one-way ANOVA followed by Tukey’s multiple-comparisons test. Dashed lines in **b**–**e** outline the spinal cord gray matter. Images are representative of two independent experiments. All images show transverse sections. Source data are provided as a source data file. Source data

Fibrosis is mediated by inflammation^4,29,30^. To investigate the recruitment of GLAST^+^ pericytes and perivascular fibroblasts in response to local inflammation, we injected lipopolysaccharide (LPS), a potent activator of monocytes/macrophages and microglia^31,32^, into the spinal cord gray or white matter of recombined *GLAST-CreER*^*T2*^;*R26R-tdTomato*;*Pdgfrb-eGFP* and *Col1a1-CreER*^*T2*^;*R26R-tdTomato*;*Pdgfrb-eGFP* mice (Fig. 6a). Focal injection of LPS resulted in comparable recruitment of MAC2^+^ macrophages/microglia and accumulation of *Pdgfrb*-EGFP^+^ stromal fibroblasts at the injection site in both gray and white matter regions (Fig. 6b–f). Interestingly, only *GLAST-CreER*^*T2*^ tdTomato^+^ cells contributed to stromal fibroblasts in the gray matter, whereas the contribution of *Col1a1-CreER*^*T2*^ tdTomato^+^ cells was negligible at 5 days after injection (Fig. 6b,c,g). In contrast, both lines contributed substantially to stromal fibroblasts after LPS injections into the white matter (Fig. 6d,e,g). The recruitment of pericytes and fibroblasts after gray and white matter LPS injections did not differ regarding cell proliferation, upregulation of *Col1a1* or detachment from the vasculature (Supplementary Fig. 9a–g). In summary, perivascular fibroblasts and GLAST^+^ pericytes are recruited in response to focal inflammation. LPS injection caused minimal tissue disruption; under these circumstances, the local recruitment of fibroblasts and pericytes was restricted to white and gray matter regions, respectively, corroborating our results after SCI (Fig. 5).

### Injury-induced myofibroblast generation

**Fig. 7:**
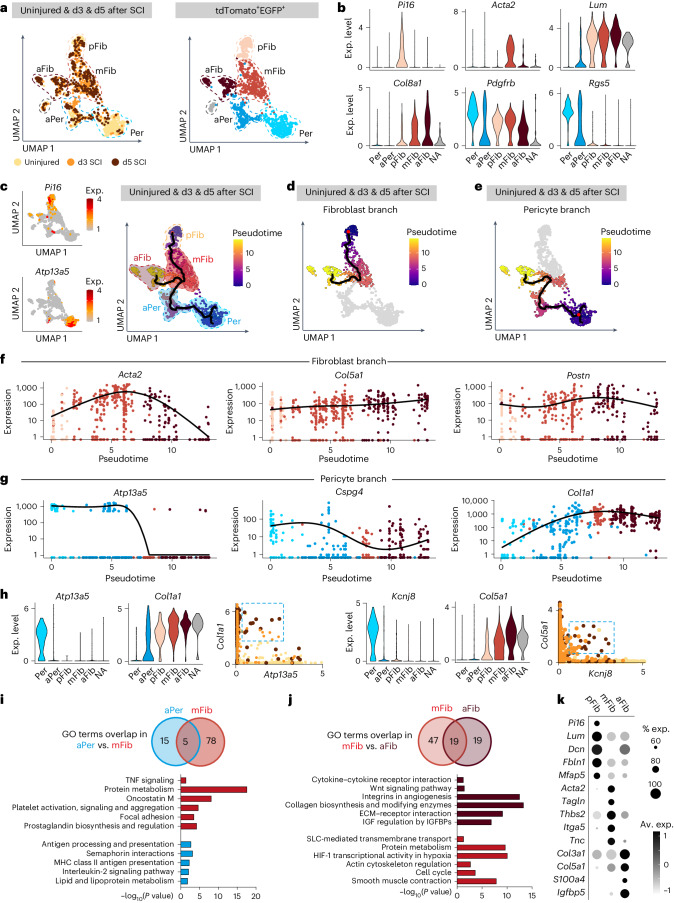
Injury induces GLAST-expressing pericyte and perivascular fibroblast activation and the generation of myofibroblasts. **a**, UMAP plot of tdTomato^+^EGFP^+^ cells isolated from the uninjured and injured spinal cord of *GLAST-CreER*^*T2*^;*R26R-tdTomato*;*Pdgfrb-eGFP* mice at 3 and 5 days after injury grouped by injury state (left) and cell population (right). **b**, Violin plots showing the expression levels of cell population-specific genes. **c**, Left, feature plots showing the expression of the fibroblast marker *Pi16* and pericyte marker *Atp13a5*. Right, pseudotime trajectory analysis of tdTomato^+^EGFP^+^ cells isolated from the uninjured and injured spinal cord at 3 and 5 days after injury. Dashed lines outline cell populations. High expression of *Pi16* and *Atp13a5* were used to identify pseudotime starting points in the fibroblast and pericyte populations, respectively. **d**,**e**, Highlight of the pseudotime trajectory of the fibroblast branch (**d**) and pericyte branch (**e**). **f**, Gene expression profile of *Acta2*, *Col5a1* and *Postn* along pseudotime within the fibroblast branch. **g**, Gene expression profile of *Atp13a5*, *Cspg4* and *Col1a1* along pseudotime within the pericyte branch. **h**, Left, violin plots showing the gene expression levels of *Atp13a5* and *Col1a1* per cell population and coexpression graph of *Col1a1* and *Atp13a5*. Right, violin plots showing the gene expression levels of *Kcjn8* and *Col5a1* per cell population and coexpression graph of *Col5a1* and *Kcnj8*. Each dot in coexpression graphs represents a single cell, colored according to the injury state. **i**,**j**, GO analyses based on upregulated genes of activated pericytes and myofibroblasts (**i**) and upregulated genes of myofibroblasts and activated fibroblasts (**j**). Venn diagrams represent the overlap of common GO terms and unique GO terms between the indicated populations. Relevant GO terms per comparison are indicated below. **k**, Dot plot of upregulated marker genes per fibroblast population. d3, day 3; d5, day 5; Per, pericyte; aPer, activated pericyte; pFib, perivascular fibroblast; mFib, myofibroblast; aFib, activated fibroblast; NA, nonattributed; TNF, tumor necrosis factor; MHC, major histocompatibility complex; IGF, insulin-like growth factor; IGFBP, insulin-like growth factor-binding protein; SLC, solute carrier; HIF-1, hypoxia-inducible factor. Source data are provided as a source data file. Source data

**Extended Data Fig. 3:**
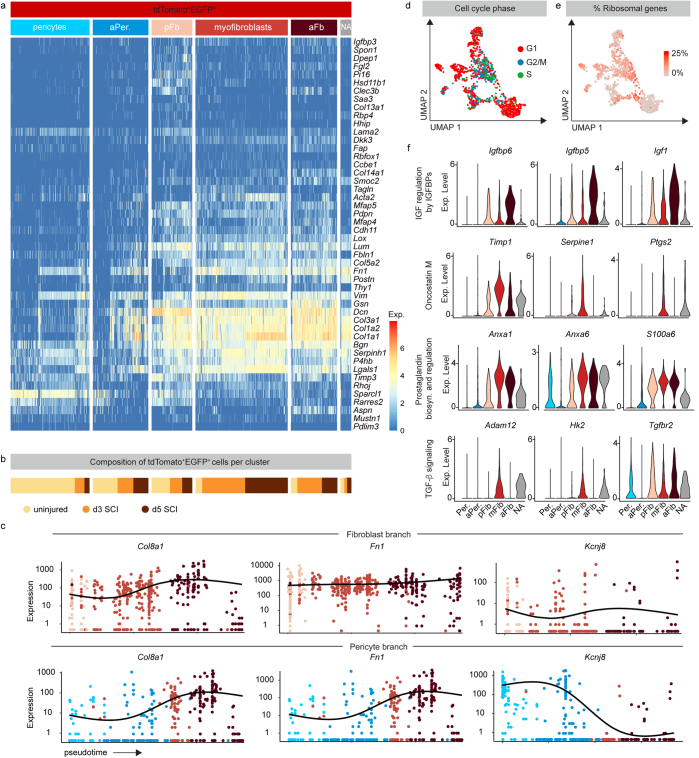
SCI induces GLAST^+^ pericyte and fibroblast activation and the generation of myofibroblasts. (**a**) Heatmap showing single-cell RNA-sequencing data of tdTomato^+^EGFP^+^ cells from the uninjured and injured spinal cord (3 and 5 days after complete spinal crush) of *GLAST-CreER*^*T2*^;*R26R-tdTomato*;*Pdgfrb-eGFP* mice subdivided by clusters based on Fig. 7a. Expression level of selected genes relevant for fibrosis are shown; scale: log counts. (**b**) Contribution of cells from different conditions (uninjured, 3 and 5 days after complete spinal crush) to each cluster (labelled in a), clustering based on Fig. 7a. (**c**) Expression of *Col8a1*, *Fn1* and *Kcnj8* genes (note scale on y-axis) along pseudotime within the fibroblast (left) and pericyte (right) branches. (**d**) UMAP plot based on Fig. 7a showing tdTomato^+^EGFP^+^ cells color-coded for cell cycle phases (G1, G2/M, S), determined with the CellCycleScoring function in Seurat. (**e**) UMAP representation of the percentage of ribosomal genes detected in comparison to total gene count, assessed with the PercentageFeatureSet function in Seurat. (**f**) Violin plots of genes relevant for GO terms based on Fig. 7i and j.

To investigate transcriptional changes underlying the fibrotic injury response, we lineage traced GLAST^+^ pericytes and fibroblasts using *GLAST-CreER*^*T2*^;*R26R-tdTomato*;*Pdgfrb-eGFP* mice and FACSorted tdTomato^+^EGFP^+^ and tdTomato^−^EGFP^+^ cells from lesion sites at 3 and 5 days after a complete spinal crush. Single cells were sequenced, and data were combined with the dataset from the uninjured spinal cord (Fig. 1). Uniform Manifold Approximation and Projection (UMAP) of the integrated data of all tdTomato^+^EGFP^+^ cells from the uninjured and injured spinal cord resulted in five connected clusters encompassing pericytes, activated pericytes, myofibroblasts, activated fibroblasts and fibroblasts (Fig. 7a,b and Extended Data Fig. 3a,b). The fibroblast marker *Pi16* (ref. ^33^) was highly expressed in perivascular fibroblasts before injury (Fig. 7b,c and Extended Data Fig. 3a). Robust *Rgs5* and *Atp13a5* expression identified pericytes in uninjured tissue (Fig. 7b,c), whereas *Acta2* expression identified myofibroblasts. All fibroblast populations expressed *Lum* and *Col8a1* (Fig. 7b)*. Col8a1* was expressed at low levels in perivascular fibroblasts before injury and highly in activated fibroblasts after injury (Fig. 7b). Next, we performed pseudotime analysis, defining two starting points: uninjured pericytes (highest *Atp13a5* expression) and uninjured fibroblasts (highest *Pi16* expression) (Fig. 7c). The analysis revealed two trajectories that merged in the myofibroblast cluster (Fig. 7c), representing the fibroblast (Fig. 7d) and pericyte (Fig. 7e) branches. The common endpoint of the two branches was activated fibroblasts (Fig. 7c–e). The fibroblast branch showed high expression of the ECM-associated genes *Fn1*, *Col5a1*, *Col8a1* and *Postn*, which gradually increased along the trajectory, whereas *Acta2* expression peaked in the myofibroblast cluster (Fig. 7f and Extended Data Fig. 3c). Along the pericyte branch, pericyte marker gene expression (*Atp13a5*, *Cspg4*, *Kcnj8*) rapidly decreased during the transition from pericytes through activated pericytes to myofibroblasts, whereas the cells simultaneously acquired a fibroblast gene expression signature (*Col1a1*, *Col8a1*, *Fn1*) (Fig. 7g and Extended Data Fig. 3c). Despite this rapid change in gene expression, we found a small population of cells that coexpressed the pericyte marker genes *Atp13a5* and *Kcnj8* (ref. ^11^) and the fibroblast markers *Col1a1* and *Col5a1*, representing a transition stage (Fig. 7h and Supplementary Fig. 10a–f). GLAST-expressing perivascular cells proliferate, detach from the blood vessel wall and migrate into the lesioned tissue^3,6^. Accordingly, a large fraction of activated pericytes and myofibroblasts expressed genes associated with the S and G_2_/M cell-cycle phases, whereas perivascular fibroblasts, pericytes and activated fibroblasts were mostly postmitotic (Extended Data Fig. 3d). With the detachment from the vascular wall, pericytes lose their pericyte identity and rapidly acquire a myofibroblast phenotype. In agreement, tdTomato^+^EGFP^+^ cells coexpressing pericyte and fibroblast markers were only sporadically detected associated with blood vessels close to the lesion, whereas *Col1a1* was highly expressed by stromal myofibroblasts in the lesion core (Supplementary Fig. 11a,b).

Activated pericytes and myofibroblasts are highly proliferative (Extended Data Fig. 3d) and commonly express genes related to transforming growth factor-β (TGFβ) regulation of ECM, brain-derived neurotrophic factor and epidermal growth factor receptor 1 signaling. GO analysis revealed 15 terms specifically enriched in activated pericytes, the most relevant of which were related to active immunity and lipid metabolism (Fig. 7i). In addition to oncostatin M and platelet activation, the GO term ‘protein metabolism’ was significantly enriched in myofibroblasts, indicative of cell activation, in line with higher expression of ribosomal genes (Fig. 7i and Extended Data Fig. 3e,f). Myofibroblasts consequently differentiated into activated fibroblasts (Fig. 7c), which showed more pronounced expression of genes related to collagen biosynthesis, cytokine interaction, angiogenesis and insulin-like growth factor-related signaling (Fig. 7j and Extended Data Fig. 3e). In turn, myofibroblasts showed higher expression of genes related to proliferation, protein metabolism, hypoxia and smooth muscle contraction. Myofibroblasts and activated fibroblasts shared GO terms related to TGFβ signaling and ECM organization (Fig. 7j and Extended Data Fig. 3e).

**Extended Data Fig. 4:**
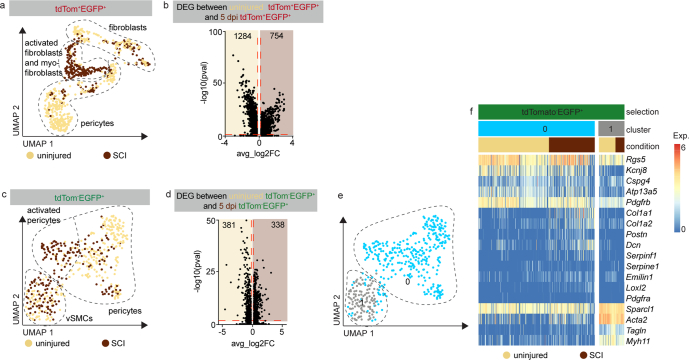
GLAST^+^ pericytes and fibroblasts and a subpopulation of GLAST^−^pericytes get activated upon SCI. (**a**) UMAP representation of tdTomato^+^EGFP^+^ cells isolated from the uninjured and injured spinal cord of *GLAST-CreER*^*T2*^;*R26R-tdTomato*;*Pdgfrb-eGFP* mice at 5 days after complete spinal crush, color-coded by injury condition. Dashed lines outline pericytes, fibroblasts, as well as activated fibroblasts and myofibroblasts based on marker expression. (**b**) Volcano plot of differentially expressed genes (DEG) between uninjured and 5 days post injury (dpi) tdTomato^+^EGFP^+^ cells. (**c**) UMAP projection of tdTomato^−^EGFP^+^ cells isolated from the uninjured and injured spinal cord of *GLAST-CreER*^*T2*^*;R26R-tdTomato;Pdgfrb-eGFP* mice at 5 days after complete spinal crush, color-coded by injury condition. Dashed lines outline pericytes, vascular smooth muscle cells (vSMCs) and activated pericytes based on marker expression. (**d**) Volcano plot of DEG between uninjured and 5 dpi tdTomato^−^EGFP^+^ cells. (**e**) UMAP color-coded for Seurat cluster analysis, separating tdTomato^−^EGFP^+^ pericytes (cluster 0) and vSMCs (cluster 1). (**f**) Heatmap of tdTomato-EGFP^+^ cells shown in (**c**-**e**) separated by cluster and injury condition, showing gene expression of selected pericyte, fibroblast, extracelullar matrix (ECM), angiogenesis, and vSMC marker genes. Activated tdTomato^−^EGFP^+^ pericytes at 5 dpi do not cluster separately (**c**), but expression of genes related to ECM and angiogenesis indicate activation of a subset of cells (**f**).

After injury, tdTomato^+^EGFP^+^ cells, which comprise pericytes and fibroblasts in the uninjured spinal cord, generated a new cell cluster of myofibroblasts and activated fibroblasts, resulting in gene expression changes in 2,038 genes (Extended Data Fig. 4a,b). In contrast, tdTomato^−^EGFP^+^ cells retained their pericyte and vSMC identity after injury, with some pericytes exhibiting an activated gene signature (Extended Data Fig. 4c). Accordingly, SCI led to expression changes in only 719 genes (Extended Data Fig. 4d). TdTomato^−^EGFP^+^ activated pericytes upregulated genes related to ECM deposition and angiogenesis and may, therefore, represent a population of pericytes that remain part of the blood vessel wall, promoting revascularization and contributing to fibrotic ECM production upon injury (Extended Data Fig. 4e,f and Supplementary Fig. 11a,b).

**Extended Data Fig. 5:**
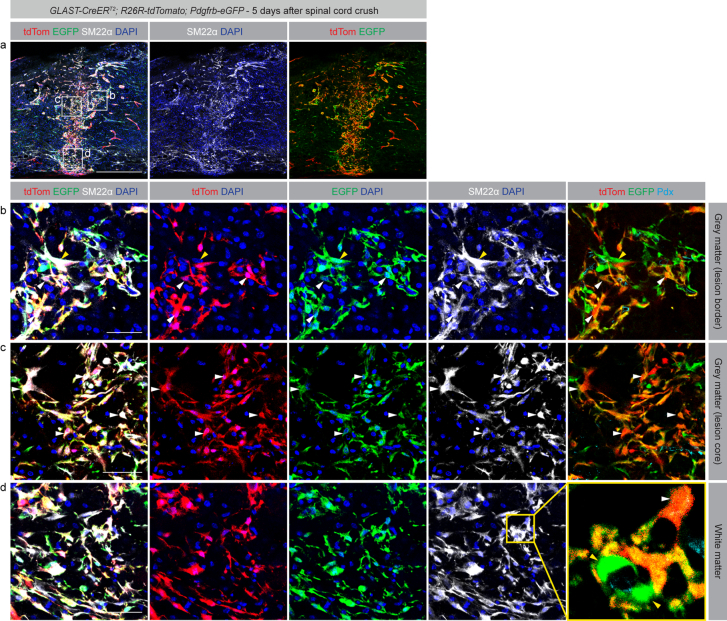
Activated pericytes, fibroblasts and myofibroblasts populate the lesion site after SCI. (**a**) Overview images of the lesion site of *GLAST*-*CreER*^*T2*^;*R26R-tdTomato*;*Pdgfrb-eGFP* mice at 5 days after complete spinal cord crush. The majority of tdTomato^+^EGFP^+^ scar-forming cells expresses high levels of smooth muscle protein 22-alpha (SM22α, gene name Tagln), a marker of myofibroblasts and vascular smooth muscle cells (vSMCs). (**b**) Close-up of the grey matter lesion border. TdTomato^−^EGFP^+^SM22α^+^ cells associated with podocalyxin^+^ (Pdx) blood vessels (vSMCs or activated pericytes; yellow arrowhead) are mainly located in the lesion border intermingled with tdTomato^+^EGFP^+^SM22α^+^ myofibroblasts (white arrowheads). (**c**) Close-up of the grey matter lesion core showing that myofibroblasts are mostly derived from tdTomato^+^EGFP^+^ perivascular cells (white arrowhead). (**d**) Close up of the lesion in the white matter. The cellular composition and distribution, and the expression level of SM22α in the white matter injury compartment is comparable to the grey matter. Magnified boxed region on the right side shows blood vessel-associated tdTomato^−^EGFP^+^SM22α^+^ vSMCs or activated pericytes surrounded by tdTomato^+^EGFP^+^SM22α^+^ myofibroblasts (white arrowhead). Scale bars: 500 μm (**a**) and 50 μm (**b**-**d**). Cell nuclei are labeled with DAPI. Images are representative of two independent experiments. All images show sagittal sections.

To differentiate perivascular fibroblasts, myofibroblasts and activated fibroblasts, we directly compared their expression of marker genes (Fig. 7k). Perivascular fibroblasts expressed elevated levels of *Pi16*, *Lum*, *Dcn*, *Fbln1* and *Mfap5*. Myofibroblasts showed strong expression of *Acta2*, *Tagln*, *Thbs2*, *Itga5* and *Tnc*, whereas activated fibroblasts displayed higher expression of *Col3a1*, *Col5a1*, *S100a4* and *Igfbp5* (Fig. 7k). Five days after SCI, most tdTomato^+^EGFP^+^ cells in the lesion expressed *Col1a1*, αSMA and SM22α, identifying them as myofibroblasts. Additionally, we identified a subset of tdTomato^+^EGFP^+^*Col1a1*^+^ cells with low αSMA and SM22α expression, which are presumably activated fibroblasts (Extended Data Fig. 5a–d and Supplementary Fig. 11a–c). VSMCs, also expressing αSMA and SM22α, were distinguished from myofibroblasts and activated fibroblasts by their lack of *Col1a1* expression (Supplementary Fig. 11b).

Together, our data show that, upon SCI, GLAST-expressing pericytes and fibroblasts give rise to myofibroblasts and activated fibroblasts, which populate the lesion and produce abundant fibrotic ECM. Activated fibroblasts and myofibroblasts show distinct fibroblast marker expression, suggesting functional differences between these stromal cell populations during CNS wound repair.

## Discussion

Fibrotic scar tissue formation is a common response to numerous CNS injuries and diseases in mice and humans^5,10^. Previous research has identified perivascular cells, including perivascular fibroblasts and GLAST-expressing perivascular cells (also known as type A pericytes), as primary contributors to stromal fibroblasts in CNS lesions^3,4,6,10,12,34–36^. Recent insights from single-cell transcriptomics data revealed that, beyond pericytes, GLAST (*Slc1a3*) is also expressed by a subset of fibroblasts in the adult mouse brain^11^, suggesting that GLAST-expressing perivascular cells may be more heterogeneous than previously assumed.

Indeed, our single-cell gene expression profiling revealed that GLAST-expressing perivascular cells can be transcriptionally defined as a subset of pericytes and a population of fibroblasts. GLAST^+^*Col1a1*^*−*^ pericytes are located in the postarteriolar transitional zone and the capillary bed; they are most abundant in gray matter regions because, anatomically, the gray matter contains a larger capillary network compared to the white matter regions of the spinal cord^37,38^. However, GLAST^+^ pericytes are distinct from classic GLAST^−^ pericytes at the ultrastructural level, exhibiting different morphological features and electron density. These observations are in line with reports describing morphological pericyte heterogeneity in the cerebral cortex^39,40^.

We observed local recruitment of pericytes and perivascular fibroblasts in response to inflammation and SCI. Perivascular fibroblasts, predominantly located around large arterioles and venules in the spinal cord white matter, contributed more to scarring in the white matter than in gray matter regions, consistent with the results of a previous study indicating the pivotal role of fibroblasts in white matter scarring following EAE^4^. However, accurately assessing the quantitative contribution of perivascular fibroblasts to fibrotic scarring in EAE may necessitate validation with an inducible *Col1a1-CreER* line, given the expression of *Col1a2* in some CNS pericytes and vSMCs^11^. The *Col1a1-CreER*^*T2*^ line used in this study recombined, on average, 81.5% of all *Col1a1*-expressing cells, with higher efficacy observed in white than in gray matter regions (Supplementary Fig. 5d), potentially underrepresenting gray matter perivascular fibroblasts and their contribution to fibrosis.

While only a small subset of perivascular cells express *Col1a1* under homeostatic conditions in the adult mouse spinal cord, we observed that virtually all scar-forming fibroblasts express *Col1a1* after SCI (Fig. 2c and Extended Data Fig. 4), corroborating previous observations with *Col1a1*-GFP reporter mice^12^. However, our lineage tracing revealed that only approximately 40% of scar-forming fibroblasts originate from *Col1a1*-expressing perivascular fibroblasts. Conversely, GLAST-expressing perivascular cells, encompassing a subset of pericytes and perivascular fibroblasts, gave rise to most scar-forming fibroblasts after SCI, aligning with previous observations^6,10^ and implying the contribution of GLAST^+^*Col1a1*^−^ pericytes to fibrotic scarring in the gray matter. This interpretation is supported by our single-cell expression analysis showing a transcriptional trajectory from GLAST^+^ pericytes to myofibroblasts and activated fibroblasts. Direct proof of a lineage relationship between GLAST^+^ pericytes and scar-forming fibroblasts is currently limited owing to the inaccessibility of inducible *CreER* lines that specifically and efficiently label the GLAST^+^ pericyte fraction. Readily available *CreER* lines targeting pericytes also label fibroblasts (*Pdgfrb-CreER*^*T2*^) or are inefficient (*NG2-CreER*^*T2*^)^12^. Another limitation is that GLAST is expressed in only a small subset of pericytes, and many pericyte markers are not homogeneously expressed by all pericytes (Fig. 1e,g and Extended Data Fig. 4f). A potential invasion of meningeal fibroblasts is rather limited in nonpenetrating CNS lesions but cannot be formally excluded. The ability of GLAST^+^ perivascular cells to generate stromal fibroblasts seems to be governed by cell-intrinsic factors, as GLAST^−^ perivascular cells sharing the same microenvironment do not contribute to stromal fibroblasts after injury. Our single-cell transcriptomics data affirm that GLAST-expressing perivascular cells give rise to fibrotic ECM-producing myofibroblasts after SCI. Pseudotime analyses suggest the transcriptional convergence of GLAST^+^ pericytes and perivascular fibroblasts toward myofibroblast generation, corroborating our previous findings regarding the role of GLAST^+^ perivascular cell-derived progeny in wound contraction and fibrotic ECM deposition, which are crucial for wound closure after SCI^3^. Notably, GLAST^+^ fibroblasts express *Pi16*, indicative of a conserved fibroblast subset signature across organs^33^. Moreover, we identified a subset of GLAST^−^ pericytes that activate a fibrotic gene signature after injury, highlighting the involvement of other perivascular cells, in addition to scar-forming myofibroblasts, in fibrotic ECM secretion.

Our results reveal that GLAST^+^ pericytes and *Col1a1*^+^ perivascular fibroblasts contribute to fibrotic scar tissue in a region-dependent manner after SCI. This emphasizes the role of locally recruited perivascular cells in fibrotic tissue formation, which may be vital for designing therapeutic strategies for fibrotic diseases.

## Methods

### Transgenic mice

#### Generation of *Col1a1-CreER*^*T2*^ transgenic mice

C57BL/6J congenic mice that express a tamoxifen-dependent Cre recombinase under the control of the *Col1a1* promoter (Tg(Col1a1-creER^T2^)6.1.Ics) were generated at PHENOMIN-Institut Clinique de la Souris, Strasbourg, France. The line was generated by microinjection of a circular murine BAC transgene (RP23-53E2) previously modified by recombineering at the ATG of the *Col1a1* gene of a rabbit β-globin intron-CreER^T2^ cassette, followed by a flipped PGK-Tn5-Kana/NeoR cassette. The genetic background of the fertilized oocytes was C57BL/6J–SJL/J. The founder was then backcrossed in a C57BL/6J genetic background. The 6.1 subline was analyzed by reverse transcription–qPCR screening and cryopreserved. Available information on the characterization of the mouse line is accessible at http://mousecre.phenomin.fr/synthesis/152. This line was deposited in the Infrafrontier repository (https://www.infrafrontier.eu/) under the identifier EM:14947.

#### Crossing of reporter lines

*GLAST-CreER*^*T2*^ (ref. ^41^) or *Col1a1-CreER*^*T2*^ mice were crossed to the *Rosa26-tdTomato* Cre reporter line^42^ (B6.Cg-Gt(ROSA)26Sor tm14(CAG-tdTomato)Hze/J, JAX stock no. 007914) to obtain *GLAST-CreER*^*T2*^;*R26R-tdTomato* or *Col1a1-CreER*^*T2*^;*R26R-tdTomato* mice, respectively. For some experiments, these mice were further crossed to the *Pdgfrb-eGFP* reporter line^43^ (Tg(Pdgfrb-EGFP)JN169Gsat/Mmucd, RRID: MMRRC 031796-UCD) to generate *GLAST-CreER*^*T2*^;*R26R-tdTomato*;*Pdgfrb-eGFP* or *Col1a1-CreER*^*T2*^;*R26R-tdTomato*;*Pdgfrb-eGFP* mice. For one experiment, *Col1a1-CreER*^*T2*^ mice were further crossed to Rasless mice^44^ to generate *Col1a1-CreER*^*T2*^;Rasless mice. All Rasless mice used were homozygous for *H-Ras* and *N-Ras* null alleles and homozygous for floxed *K-Ras* alleles; Cre^−^ mice were used as controls.

All strains were backcrossed to the C57BL/6J genetic background. Both male and female mice aged >8 weeks were included in the experiments, except when stated otherwise. All animals were housed in groups and kept in standardized individually ventilated cages (Scanbur), with controlled humidity (50%) and temperature (22 °C) and a 12-h/12-h light/dark cycle with water and food ad libitum. All experimental procedures were carried out in accordance with the Swedish and European Union law and guidelines and approved by the regional ethical committee (Stockholm Ethical Committee/‘Stockholms Djurförsöksetiska Nämnd’). Based on their genotype, mice were randomly assigned to the experimental groups.

### Genetic labeling of transgenic mice

Recombination of *CreER*^*T2*^ transgenic lines was induced by a daily intraperitoneal (i.p.) injection of 2 mg of tamoxifen (Sigma-Aldrich, T5648, 20 mg ml^−1^ in 9:1 corn oil/ethanol) for five consecutive days, followed by a 7-day clearing period^28^.

### Surgical procedures

For all injury models, mice were anesthetized with 4% isoflurane until sedated, followed by 2% isoflurane during surgery. All animals received preemptive and postoperative pain relief (Temgesic (buprenorphine), Schering-Plough, 0.1 mg kg^−1^ body weight and Rimadyl (carprofen), Pfizer, 5 mg kg^−1^ body weight; subcutaneous injection), as well as local anesthesia before spinal lesions or intraspinal injections (Xylocaine (lidocaine), AstraZeneca, 10 mg ml^−1^ and Marcaine (bupivacaine), AstraZeneca, 2 mg kg^−1^ body weight). Viscotears (carbomer) gel (2 mg g^−1^, Novartis) was topically applied to prevent the eyes from drying out. After surgery, the animals were placed in a prewarmed cage under close observation and transferred to their home cage thereafter.

#### Complete spinal cord crush

A laminectomy was conducted to expose the spinal cord at T10. After the application of local anesthesia, the spinal cord was fully crushed for 2 s with Dumont No. 5 forceps (11295-00, Fine Science Tools)^10,45^. Animals received postoperative analgesia as described above and were treated with antibiotics (Hippotrim vet., sulfadiazine 200 mg ml^−1^, trimethoprim 40 mg ml^−1^, 100 mg kg^−1^ body weight every 24 h; subcutaneous injection) to prevent bladder infection. To help the animals’ mobility, we supplemented the cage floor with a raised grid. Throughout the experiment, bladders were manually expressed two to three times per day to permit micturition. From 1 day before the surgery, the animals’ diet was supplemented with a high-energy nutritional supplement (DietGel Boost, Clear H2O) during the first week after surgery to support recovery and limit weight loss. The body weight was recorded before surgery, daily during the first week after injury and weekly thereafter. Only female mice were used for crush lesion experiments. Mice were killed at 3, 5, 7 and 14 days after injury.

#### Spinal cord contusion

For spinal cord contusion lesions, mice were placed on a stabilization platform for surgery. After laminectomy, the vertebral column was stabilized at T8 and T10 vertebrae with adjustable fine pitch forceps connected to three-joint surgery arms. After the application of local anesthesia, the mice received a 40-kdyn contusion centered on the T9 thoracic spinal cord using the Infinite Horizon impactor (IH-0400, PSI) with a 1.3-mm tip. Subsequently, the wounds were sutured, and mice were placed in a preheated cage for recovery and thereafter transferred to their home cage with an elevated floor grid. Bladders were manually expressed two to three times per day to permit micturition until mice regained bladder control (usually within 5 days after surgery in this mild contusion model). See above for postoperative analgesia.

Only female mice were used for contusion lesion experiments. Mice were killed at 5 and 14 days after injury.

#### Stereotactic injection of LPS into the spinal cord

LPS (from *Escherichia coli*, ALX-581-007, Enzo Life Sciences, 1 mg ml^−1^) was stereotactically injected into the low thoracic region (T12/T13) of the mouse spinal cord in deeply anesthetized uninjured adult animals. Mice were placed on a stabilization platform for surgery. After laminectomy, the vertebral column was stabilized with adjustable fine pitch forceps connected to three-joint surgery arms. Each mouse received three injections spaced 3 mm apart, with each injection delivering a total volume of 0.2 μl (200 ng of LPS per injection) using a 10-μl NanoFil syringe with a 36-gauge beveled needle tip (World Precision Instruments) coupled to a microinjector (UltraMicroPump III and Micro4 microsyringe pump controller, World Precision Instruments). After injection, the needle was left in place for an additional 2 min to allow diffusion and prevent backflow of the solution and then slowly withdrawn. Injections were carried out 0.35-mm lateral relative to the posterior median spinal vein and at 0.5-mm depth from the spinal cord surface to inject into the gray matter or at 1.3-mm depth to inject into the ventral white matter of the spinal cord. Both male and female mice were used for intraspinal injections. Mice were killed at 5 days after injection.

### Isolation of different perivascular cell populations from the spinal cord

Preparation of single-cell cell suspensions and myelin removal were performed either using an adult brain dissociation kit (130-107-677, Miltenyi), followed by the Smart-seq3 and Smart-seq3xpress library preparation protocols, or adapted from previously published protocols, followed by the Smart-seq2 protocol^45,46^. In summary, *GLAST-CreER*^*T2*^;*R26R-tdTomato*;*Pdgfrb-eGFP* animals (uninjured and 3 and 5 days after spinal crush) were killed by i.p. injection of sodium pentobarbital (200 mg kg^−1^, 100 μl i.p., APL) and transcardially perfused with cold Hank’s balanced salt solution (HBSS) without calcium or magnesium (Invitrogen). The spinal cord was carefully dissected out of the vertebrae, and the crush injury segment was separated from the uninjured spinal cord tissue and treated separately thereafter. After the removal of the outer meningeal layers, the tissue was mechanically dissociated with a razor blade on a glass Petri dish and immediately transferred to cold HBSS. This step was followed by enzymatic digestion. Before Smart-seq2 library preparation, tissues were digested with papain at a final concentration of 8 U ml^−1^ with 80 Kunitz units of DNase I per ml (Sigma-Aldrich, D4263) in Ca^2+^/Mg^2+^-free piperazine-*N*,*N*′-bis(2-ethanesulfonic acid)/cysteine-based buffer (pH 7.4) for 40 min at 37 °C, 350 rpm on a ThermoMixer (Eppendorf). The tissue was carefully triturated and incubated for an additional 5 min. The cell suspension was passed through a 70-μm cell strainer (Corning), washed with minimum essential medium (MEM) with 1% BSA and spun down at 200*g* for 5 min at 4 °C. The cells were resuspended in MEM and centrifuged over a 90% Percoll gradient (GE Healthcare, 17-0891-01) at 250*g* for 15 min at 4 °C. Cells in the lipid layer and below were diluted five times in MEM with 1% BSA and centrifuged in a 15-ml tube at 250*g* for 10 min at 4 °C. All supernatants, including the lipid layer, were carefully removed. Before Smart-seq3 and Smart-seq3xpress library preparation, cells were enzymatically digested following the manufacturer’s instructions from the adult brain dissociation kit. Briefly, cells were incubated with enzymes for 15 min at 37 °C with agitation and then triturated by pipetting up and down with 1,000-μl tips and incubated for 10 min more. Digestion was stopped by adding 10 ml of MACS buffer (PBS with 0.5% FBS). Cells were filtered through a 70-μm cell strainer and centrifuged for 10 min at 300*g* at 4 °C.

Following the two protocols, pellets were resuspended in cold MACS buffer and magnetic myelin removal beads (Miltenyi Biotech, 130-096-433) and incubated for 15 min at 4 °C. The cells were washed and run over MACS magnetic separation columns (Miltenyi Biotech, 130-042-201) on a magnetic stand according to the manufacturer’s instructions. The cells in the flow through were collected, spun down, resuspended in FACS buffer (2% FBS in PBS) and kept on ice until further processing. For Smart-seq3xpress, cells were resuspended in pure PBS to avoid serum contamination in the lysis mix.

### Single-cell sorting, library preparation, sequencing and quality control

The single-cell suspensions generated from *GLAST-CreER*^*T2*^;*R26R-tdTomato*;*Pdgfrb-eGFP* animals were subjected to FACS. Based on reporter gene expression, tdTomato^+^EGFP^+^ and tdTomato^−^EGFP^+^ cells were collected into 96- or 384-well plates with cell lysis buffer (Clonetech, 635013) or Smart-seq3/Smart-seq3xpress lysis buffer mix, followed by a brief centrifugation (1 min, 1,500 rpm) and stored at −80 °C until further processing. Cell sorting was performed on a FACSAria III cell sorter (BD Biosciences). Singlet discrimination was performed using plots for forward scatter (FSC-A versus FSC-H) and side scatter (SSC-W versus SSC-H), and dead cells were excluded by SYTOX Blue dead cell staining (Thermo Fisher Scientific, S34857). Single cells were processed to cDNA libraries according to the Smart-seq2, Smart-seq3 or Smart-seq3xpress protocols^16–18^. Smart-seq2 samples were sequenced on Illumina HiSeq 2500 (HiSeq Control Software 2.2.58/RTA 1.18.64) with a 1 × 51 setup using ‘HiSeq SBS Kit v4’ chemistry. Smart-seq3 and Smart-seq3xpress libraries were sequenced on an Illumina NextSeq 500 (Illumina NextSeq Control Software 2.2.0). Reads were then aligned to the mouse reference genome.

### Single-cell gene expression analysis

All data were processed using Seurat (v4.3.0.1)^47^ and RStudio (v1.4.1717)^48^. Data from Smart-seq2, Smart-seq3 or Smart-seq3xpress sequencing were first preprocessed individually using Seurat package default parameters, with the first 20 principal components and 0.5 as the resolution. Cells expressing between 200 and 1,200 features and <20% of mitochondrial genes were retained for the analysis. The different datasets were then merged and integrated with Harmony (v0.1.1)^49^. To correct potential batch effects, we performed scaling with the feature number and percentage of mitochondrial and ribosomal content regression. Unbiased clustering was done using the first 30 principal components and 0.5 as the resolution. Astrocytes and immune cell contamination were removed by filtering data based on *Gfap* and *Ptprc* gene expression, respectively. Endothelial cells were removed based on high expression of *Pecam1* and low expression of *Pdgfrb*.

Differential gene expression analyses were done using the Wilcoxon test, and genes with *P* < 0.05 and log_2_(fold change) > 0.25 were used for subsequent GO analyses. GO term analyses were done using Enrichr^50^. GO functions with fewer than five genes and a *P* value of >0.05 were excluded, and analyses were performed using the BioPlanet library.

Pseudotime analyses were performed using Monocle3 (v1.3.1). Normalized data from the Seurat object were used as input for pseudotime analyses and were processed as described in the Monocle3 tutorial. To decipher the pseudotime trajectory, we first used the entire dataset by defining two starting points using the *Pi16* expression for the fibroblast branch and the *Rgs5* expression for the pericyte branch. The two branches were then divided manually using the choose_graph_segments function, and gene expression was plotted using the plot_genes_in_pseudotime function. UMAPs for the comparison of day 0 and day 5 SCI tdTomato^+^EGFP^+^ or tdTomato^−^EGFP^+^ datasets were generated using the first 15 principal components. A cluster of *Slc1a3*^+^*Pdgfra*^+^ fibroblasts in the tdTomato^−^EGFP^+^ dataset (nonrecombined GLAST^+^ cells) was excluded from the analysis. Heat maps were generated with the pheatmap package^51^.

For cell-type comparison, our dataset from the uninjured spinal cord was integrated with data from uninjured brain mural cells from ref. ^11^ (GSE98816) using the Seurat IntegrateData function. Both datasets were preprocessed by selecting cells with >350 features and <10% mtRNA, in which only genes present in more than three cells were considered. For normalization and variance stabilization, we used sctransform^52^. For plots with gene expression levels, only mural cells (pericytes, vSMCs)^11^ were included and shown as UMAP projections of the first 15 dimensions (determined based on elbow plots) and a resolution of 0.5. For analysis, we used the Seurat standard workflow, UMAP dimension reduction with 15 dimensions (determined based on elbow plots) and a resolution of 0.5. Cells annotated as endothelial cells were removed before data integration^52^.

### Tissue processing and immunohistochemistry

Animals were killed by i.p. injection of an overdose of sodium pentobarbital and transcardially perfused with cold PBS, followed by 4% formaldehyde in PBS. Spinal cords were dissected out and postfixed in 4% formaldehyde in PBS overnight at 4 °C and then cryoprotected in 30% sucrose. After embedding in OCT mounting medium and freezing on dry ice, spinal cords were sectioned with a cryostat (Leica, CM1860). Cryosections (20 μm) were then collected on alternating slides and stored at –20 or –80 °C. For immunostaining, sections were incubated with blocking solution (10% normal donkey serum in PBS, with 0.3% Triton X-100) for 1 h at room temperature and then incubated at room temperature overnight in a humidified chamber with primary antibodies diluted in 10% normal donkey serum. The primary antibodies used were GFP (1:2,000, chicken, Aves Labs, GFP-1020; 1:2,000, sheep, Bio-Rad, 4745-1051), RFP (red fluorescent protein; 1:250, chicken, Novus Biologicals, NBP1-97371), PDGFRβ (1:200, rabbit, Abcam, ab32570; 1:100, rat, eBioscience, 14-1402-82), podocalyxin (1:200, goat, R&D Systems, AF1556), αSMA (1:200, rabbit, Abcam, ab5694), SM22α (also known as transgelin; 1:500, rabbit, Abcam, ab14106), Ki67 (1:2,000, rat, eBioscience, 14-5698), NG2 chondroitin sulfate proteoglycan (1:200, rabbit, Millipore, AB5320; no Triton X-100) and MAC2 (also known as galectin-3; 1:500, rat directly conjugated to biotin, Cedarlane Labs, CL8942B).

All secondary antibodies used for immunohistochemistry were F(ab′)2 fragment affinity-purified antibodies purchased from Jackson Immunoresearch and diluted at 1:500: Alexa Fluor 488 donkey anti-chicken immunoglobulin Y (IgY) (703-546-155), Cy3 donkey anti-chicken IgY (703-166-155), Alexa Fluor 647 donkey anti-goat IgG (705-606-147), Alexa Fluor 594 donkey anti-goat IgG (705-585-147), Alexa Fluor 680 donkey anti-goat IgG (705-625-147), Alexa Fluor 488 donkey anti-rabbit IgG (711-546-152), Alexa Fluor 647 donkey anti-rabbit IgG (711-606-152), Alexa Fluor 680 donkey anti-rabbit IgG (771-625-152), Alexa Fluor 488 donkey anti-rat IgG (712-546-153), Alexa Fluor 647 donkey anti-rat IgG (712-606-153) and Alexa Fluor 488 donkey anti-sheep IgG (713-546-147). Biotinylated secondary antibodies were revealed with Alexa Fluor 594-conjugated streptavidin (1:500, Jackson Immunoresearch, 016-580-084) or Alexa Fluor 680-conjugated streptavidin (1:500, Jackson Immunoresearch, 016-620-084).

Cell nuclei were visualized with DAPI (1 μg ml^−1^ in PBS, Sigma-Aldrich, D9542). Sections were mounted using VECTASHIELD antifade mounting medium (Vector Labs, H-1000).

### In situ hybridization—RNAscope

For the detection of RNA molecules, we performed RNAscope on formaldehyde-fixed spinal cord cryosections stored at –80 °C. In situ hybridization was performed following a modified version of the RNAscope Multiplex Fluorescent Reagent Kit v2 Assay (ACD Bio-Techne, 323100). Briefly, tissue sections were allowed to dry and equilibrate to room temperature, washed in PBS and baked for 30 min at 60 °C before postfixation in 4% formaldehyde in PBS for 15 min at 4 °C. Slides were subsequently dehydrated in 50%, 70% and 100% ethanol for 5 min each at room temperature. After drying, slides were first boiled for 10 s in distilled water, followed by 5 min in antigen retrieval solution, washed in distilled water and 100% ethanol at room temperature, and allowed to dry again. For further antigen accessibility, sections were incubated with protease III (ACD Bio-Techne, 322337) at 40 °C for 30 min. The samples were washed in distilled water and incubated at 40 °C for 2 h with the following RNAscope probes (ACD Bio-Techne): Pdgfrb (Mm-Pdgfrb-C3, 411381-C3), Pdgfra (Mm-Pdgfra, 480661), Col1a1 (Mm-Col1a1, 319371), Slc1a3 (Mm-Slc1a3-C3, 430781-C3), Atp13a5 (Mm-Atp13a5-C2, 417211-C2) and Gjb6 (Mm-Gjb6, 458811). C3 probes were diluted 1:50 in C1 probes or probe diluent. Subsequent amplification and detection were performed following the assay protocol. Probes were detected with Opal dyes 520 (Perkin Elmer, FP1487A), 570 (Perkin Elmer, FP1488A) or 650 (Perkin Elmer, FP1496A). TdTomato labeling was recovered by immunohistochemistry with an antibody against RFP (1:250, chicken, Novus Biologicals, NBP1-97371). Nuclei were stained with DAPI (1:5,000, 1 μg ml^−1^ in PBS, Sigma-Aldrich, D9542) before mounting the slides. The protocol was run in 1 day to preserve sample quality.

### Light-sheet fluorescence microscopy

#### Blood vessel labeling

Animals were first transcardially perfused with cold PBS and then with 4% formaldehyde in PBS, followed by 5 ml of a 2% gelatin (Sigma-Aldrich, G1890) solution in PBS containing 0.5 mg of BSA conjugated to Alexa Fluor 647 (Thermo Fisher Scientific, A34785). Subsequently, perfused mice were kept in ice-cold water for at least 15 min for the gelatin to solidify. The labeled tissue was kept in 4% formaldehyde in PBS for further histological processing.

#### Optical tissue clearing

The tissue clearing protocol was adapted from ref. ^53^. After fixation with 4% formaldehyde in PBS for 3 days, spinal cords were optically cleared with 4% SDS at 50 °C for 3–5 days in a rotating hybridization incubator with daily renewal of the clearing solution. The cleared spinal cords were embedded in 4% agarose and washed with PBS with 0.1% Tween 20 detergent (PBS-T) overnight. The embedded spinal cords were incubated with primary antibodies against SM22α (1:500, rabbit, Abcam, ab14106) and vWF (1:100, sheep, Abcam, ab11713) diluted in PBS-T for 2 days at room temperature. After being washed with PBS-T overnight, samples were incubated with species-specific secondary antibodies conjugated to Alexa Fluor 488 (1:200, Jackson ImmunoResearch) in PBS-T for 2 days. The spinal cords were then washed and stored in PBS-T at room temperature until imaging.

#### Light-sheet imaging of cleared tissues

The embedded cleared tissues were incubated in a customized refractive index (RI) matching medium (Omnipaque 350, GE Healthcare) supplemented with 1 M urea (Sigma-Aldrich, U5378) and 40% d-sorbitol (Sigma-Aldrich, S1876; RI = 1.48) overnight. After RI matching, the samples were imaged with a light-sheet microscope (Zeiss Z.1). The acquired images were processed to achieve an isotropic resolution of 2.5 μm and visualized with napari^54^.

#### Quantitative assessment of cleared tissues

To quantify the percentage of tdTomato^+^ cells in different vessel compartments and between gray matter and white matter, a 500-μm-thick uninjured thoracic spinal cord segment was used for each sample. Counting was done manually in an interactive 3D view, assisted by a custom-built napari plug-in. The classification of arterioles, venules and capillaries was based on the SM22α and vWF signals and the relative position and blood vessel size, which were determined together by two observers.

### Electron microscopy

#### Transcardial perfusion

Uninjured *GLAST-CreER*^*T2*^;*R26R-tdTomato* and *Col1a1-CreER*^*T2*^;*R26R-tdTomato* animals were killed by i.p. injection of an overdose of sodium pentobarbital and initially transcardially perfused with ice-cold 2% dextran in 0.1 M phosphate buffer (20 s), followed by a mixture of 4% formaldehyde and 0.1% glutaraldehyde in 0.1 M phosphate buffer for 15 min. The spinal cords were removed, left in the fixative solution overnight and stored in a 1:10 dilution of the same solution in 0.1 M phosphate buffer.

#### Postembedding and immunogold electron microscopy

Small blocks were dissected out from perfusion-fixed spinal cords and subjected to a freeze-substitution procedure as described previously^55^. Briefly, tissue blocks were cryoprotected in 4% glucose overnight, and the tissues were suspended in graded glycerol solutions (10%, 20% and 30% glycerol in 0.1 M phosphate buffer for 30 min in each gradient). After cryoprotection, the tissue blocks were rapidly frozen in propane cooled to −170 °C using liquid nitrogen before being subjected to freeze substitution. Samples were later embedded in methacrylate resin (Lowicryl HM20) and polymerized by ultraviolet irradiation below 0 °C. Serial sections were cut at 90–100 nm using an ultramicrotome (Reichert Ultracut S, Leica) and placed on 300-mesh Ni grids until further use.

Immunogold cytochemistry was performed on every tenth ultrathin section as previously described^56^, and the remaining serial sections were only counterstained as described below. Briefly, the ultrathin sections were incubated with a primary antibody against RFP, which recognizes tdTomato (1:100, rabbit, Rockland, 600-401-379s), overnight at room temperature in a humidified chamber. The following day, the sections were washed and incubated with a secondary antibody (1:20, goat anti-rabbit IgG conjugated to 12-nm gold particles, Abcam, ab105298) for 2 h. The sections were counterstained using 2% uranyl acetate and 0.3% lead citrate for 90 s each. Images were acquired at 80 kV using a Tecnai 12 electron microscope (FEI Company) equipped with ITEM FEI version 5.1 software (Olympus Soft Imaging Solutions). Colored hue was added to images with Adobe Illustrator CS6 version 16.0 (Adobe Systems) to highlight cells of interest.

#### 3D modeling

The open-source software Reconstruct^57^ was used for 3D modeling. Consecutive immunogold electron microscopy images were traced to create the 3D reconstruction. All electron microscopy images used for the 3D modeling were taken with the same magnification to ease the calibration and alignment process. First, images were aligned in a semiautomatic manner using three to four corresponding reference points on two consecutive images, and this was repeated through the entire stack. Care was taken to ensure proper alignment and to avoid potential artifacts in the alignment process. Next, the structures of interest were traced using the wildfire mode, and all traces were inspected and corrected manually where needed. Rendering of the 3D model was done with the following software parameters: Generate; Botsonian surface, Normals; Vertex, Facets; 32. To unwrap selected cells from the endothelial tube, we exported isolated 3D models to the open-source software Blender by the Blender Foundation. Within Blender, cloth physics was applied to the isolated model, and the 3D model fell into the desired position.

### Imaging and quantitative analysis

Images were acquired with a Leica TCS SP8X confocal microscope equipped with LAS X 3.5.7.23225 software. Image processing and assembly were performed with ImageJ/Fiji (v2.1.0/1.53c for Mac) and Adobe Illustrator (v25.04.1 for Mac).

Transverse (Figs. 2c and 6 and Supplementary Figs. 5, 9 and 10) or sagittal (Fig. 5, Extended Data Figs. 2 and 5, and Supplementary Figs. 8 and 11) spinal cord sections, spanning the injury site and 400-μm rostral and caudal to the injury site, were collected on 20 alternating slides at 20-μm thickness and used for quantifications. Matched segments of uninjured spinal cords were sectioned and collected similarly. All quantifications were done in at least three alternate sections per animal covering the lesion epicenter and spaced 400 μm apart. Except for Extended Data Fig. 2c, data collection and analysis were not performed blind to the conditions of the experiments.

The percentages of stromal PDGFRβ^+^ cells that express tdTomato (*GLAST-CreER*^*T2*^;*R26R-tdTomato* or *Col1a1-CreER*^*T2*^;*R26R-tdTomato* mice) upon spinal cord crush or contusion and upon intraspinal injection of LPS were assessed by manual counting. The number of tdTomato^+^PDGFRβ^+^ was divided by the total number of PDGFRβ^+^ cells in the lesion core (delimitated by the glia limitans). Only cells dissociated from the blood vessel wall within the lesion core (immunostained for the endothelial markers CD31 or podocalyxin) were considered. For gray and white matter comparisons, the percentages of recombined stromal cells were determined for gray and white matter regions separately. The area covered by the MAC2^+^ signal was thresholded and measured using ImageJ/Fiji software, and averaged values are presented as the percentage of MAC2^+^ area per hemisection. The recombination efficacy in *Col1a1-CreER*^*T2*^;*R26R-tdTomato* mice was determined by the ratio of tdTomato^+^*Col1a1*^+^ cells out of the total number of cells with *Col1a1*^+^ mRNA clusters (in situ hybridization with the Mm-Col1a1 RNAscope probe). The ratio was determined in transverse spinal cord tissue sections (*n* = 6 mice) in the gray matter and white matter. The ratio for the meninges was determined within an optical field spanning one-quarter of the meninges surrounding transverse sections.

The number of *GLAST-CreER*^*T2*^ tdTomato^+^ or *Col1a1-CreER*^*T2*^ tdTomato^+^ perivascular (PDGFRβ^+^) cells in the uninjured mouse spinal cord was counted and calculated over nine transverse sections (20-μm-thick) spanning three different thoracic spinal cord segments. To determine the expression level of *Col1a1* mRNA in *GLAST-CreER*^*T2*^;*R26R-tdTomato* mice, nuclei of recombined (tdTomato^+^) cells expressing *Pdgfrb*^+^ mRNA were outlined (*n* = 4 mice, three tissue sections each) and the integrated density of the *Col1a1* signal was measured using ImageJ/Fiji software. The analyzed cells were divided into tdTomato^+^ cells occupying large-caliber (>6-μm inner diameter) or small-caliber (<6-μm inner diameter) blood vessels, mainly in the gray matter of the spinal cord. The blood vessels were labeled with *Lycopersicon esculentum* lectin (B1175, Vector Laboratories).

### Statistical analysis

Sample sizes were determined based on previous experience^3,10^. Data are presented as mean ± s.d., and individual data points are plotted in the graphs. The number of animals (sample size) and statistical tests used are noted in the figure legends. Normality tests were used to assess the Gaussian distribution of the datasets, followed by appropriate statistical tests. Statistical analyses were done with GraphPad Prism (v9.3.1). *P* values were calculated using two-tailed unpaired Student’s *t* test and one-way ANOVA followed by Šidáks or Tukey’s multiple-comparisons test. Differences were considered statistically significant when *P* < 0.05. Source data are provided as source data files.

### Reporting summary

Further information on research design is available in the Nature Portfolio Reporting Summary linked to this article.

## Online content

Any methods, additional references, Nature Portfolio reporting summaries, source data, extended data, supplementary information, acknowledgements, peer review information; details of author contributions and competing interests; and statements of data and code availability are available at 10.1038/s41593-024-01678-4.

### Supplementary information

Supplementary Information Supplementary Figs. 1–11.

Reporting Summary

Supplementary Data Source data for Supplementary Figs. 4–6 and 9.

### Source data

Source Data Fig. 2 Individual data points/counted values.

Source Data Fig. 5 Individual data points/counted values.

Source Data Fig. 6 Individual data points/counted values.

Source Data Fig. 7 Full list of GO terms.

Source Data Extended Data Fig. 1 Individual data points/counted values.

Source Data Extended Data Fig. 2 Individual data points/counted values.

## Data Availability

The authors declare that all data supporting the findings of this study are included in this published article and its supplementary information files. The publicly available dataset GSE98816 was used for comparisons in Extended Data Fig. 2, and the mouse reference genome used was GCF_000001635.20/27. Single-cell RNA-sequencing data are deposited in the Gene Expression Omnibus (GEO) database (accession nos. GSE229916, GSE266250 and GSE266251). Source data are provided with this paper.

## References

[1] Ruschel J (2015). Axonal regeneration. Systemic administration of epothilone B promotes axon regeneration after spinal cord injury. Science.

[2] Brazda N, Müller HW (2009). Pharmacological modification of the extracellular matrix to promote regeneration of the injured brain and spinal cord. Prog. Brain Res..

[3] Dias DO (2018). Reducing pericyte-derived scarring promotes recovery after spinal cord injury. Cell.

[4] Dorrier CE (2021). CNS fibroblasts form a fibrotic scar in response to immune cell infiltration. Nat. Neurosci..

[5] Dias DO, Göritz C (2018). Fibrotic scarring following lesions to the central nervous system. Matrix Biol..

[6] Göritz C (2011). A pericyte origin of spinal cord scar tissue. Science.

[7] Hellal F (2011). Microtubule stabilization reduces scarring and causes axon regeneration after spinal cord injury. Science.

[8] Yoshioka N, Hisanaga S, Kawano H (2010). Suppression of fibrotic scar formation promotes axonal regeneration without disturbing blood–brain barrier repair and withdrawal of leukocytes after traumatic brain injury. J. Comp. Neurol..

[9] Zhu Y (2015). Hematogenous macrophage depletion reduces the fibrotic scar and increases axonal growth after spinal cord injury. Neurobiol. Dis..

[10] Dias DO (2021). Pericyte-derived fibrotic scarring is conserved across diverse central nervous system lesions. Nat. Commun..

[11] Vanlandewijck M (2018). A molecular atlas of cell types and zonation in the brain vasculature. Nature.

[12] Soderblom C (2013). Perivascular fibroblasts form the fibrotic scar after contusive spinal cord injury. J. Neurosci..

[13] Lendahl U, Nilsson P, Betsholtz C (2019). Emerging links between cerebrovascular and neurodegenerative diseases—a special role for pericytes. EMBO Rep..

[14] Armulik A, Genové G, Betsholtz C (2011). Pericytes: developmental, physiological, and pathological perspectives, problems, and promises. Dev. Cell.

[15] Sofroniew MV (2021). Inflammation drives fibrotic scars in the CNS. Nat. Neurosci..

[16] Hagemann-Jensen M, Ziegenhain C, Sandberg R (2022). Scalable single-cell RNA sequencing from full transcripts with Smart-seq3xpress. Nat. Biotechnol..

[17] Hagemann-Jensen M (2020). Single-cell RNA counting at allele and isoform resolution using Smart-seq3. Nat. Biotechnol..

[18] Picelli S (2014). Full-length RNA-seq from single cells using Smart-seq2. Nat. Protoc..

[19] McKenzie AT (2018). Brain cell type specific gene expression and co-expression network architectures. Sci. Rep..

[20] Marques S (2016). Oligodendrocyte heterogeneity in the mouse juvenile and adult central nervous system. Science.

[21] Zamboni M, Llorens-Bobadilla E, Magnusson JP, Frisén J (2020). A widespread neurogenic potential of neocortical astrocytes is induced by injury. Cell Stem Cell.

[22] De Bock M (2014). A new angle on blood–CNS interfaces: a role for connexins?. FEBS Lett..

[23] Hirschi KK, Burt JM, Hirschi KD, Dai C (2003). Gap junction communication mediates transforming growth factor-β activation and endothelial-induced mural cell differentiation. Circ. Res..

[24] Ivanova E, Kovacs‐Oller T, Sagdullaev BT (2019). Domain‐specific distribution of gap junctions defines cellular coupling to establish a vascular relay in the retina. J. Comp. Neurol..

[25] Mazaré N, Gilbert A, Boulay A-C, Rouach N, Cohen-Salmon M (2018). Connexin 30 is expressed in a subtype of mouse brain pericytes. Brain Struct. Funct..

[26] Lendahl U, Muhl L, Betsholtz C (2022). Identification, discrimination and heterogeneity of fibroblasts. Nat. Commun..

[27] Krueger M, Bechmann I (2010). CNS pericytes: concepts, misconceptions, and a way out. Glia.

[28] Meletis K (2008). Spinal cord injury reveals multilineage differentiation of ependymal cells. PLoS Biol..

[29] Lee SB, Kalluri R (2010). Mechanistic connection between inflammation and fibrosis. Kidney Int. Suppl..

[30] Mack M (2018). Inflammation and fibrosis. Matrix Biol..

[31] Felts PA (2005). Inflammation and primary demyelination induced by the intraspinal injection of lipopolysaccharide. Brain.

[32] Batista CRA, Gomes GF, Candelario-Jalil E, Fiebich BL, de Oliveira ACP (2019). Lipopolysaccharide-induced neuroinflammation as a bridge to understand neurodegeneration. Int. J. Mol. Sci..

[33] Buechler MB (2021). Cross-tissue organization of the fibroblast lineage. Nature.

[34] Eilken HM (2017). Pericytes regulate VEGF-induced endothelial sprouting through VEGFR1. Nat. Commun..

[35] Liu J (2021). A human cell type similar to murine central nervous system perivascular fibroblasts. Exp. Cell Res..

[36] Milich LM (2021). Single-cell analysis of the cellular heterogeneity and interactions in the injured mouse spinal cord. J. Exp. Med..

[37] Martirosyan NL (2011). Blood supply and vascular reactivity of the spinal cord under normal and pathological conditions. J. Neurosurg. Spine.

[38] Kirst C (2020). Mapping the fine-scale organization and plasticity of the brain vasculature. Cell.

[39] Grant RI (2019). Organizational hierarchy and structural diversity of microvascular pericytes in adult mouse cortex. J. Cereb. Blood Flow. Metab..

[40] Hartmann DA (2015). Pericyte structure and distribution in the cerebral cortex revealed by high-resolution imaging of transgenic mice. Neurophotonics.

[41] Slezak M (2007). Transgenic mice for conditional gene manipulation in astroglial cells. Glia.

[42] Madisen L (2010). A robust and high-throughput Cre reporting and characterization system for the whole mouse brain. Nat. Neurosci..

[43] Gong S (2003). A gene expression atlas of the central nervous system based on bacterial artificial chromosomes. Nature.

[44] Drosten M (2010). Genetic analysis of Ras signalling pathways in cell proliferation, migration and survival. EMBO J..

[45] Lovatt D (2007). The transcriptome and metabolic gene signature of protoplasmic astrocytes in the adult murine cortex. J. Neurosci..

[46] Orre M (2014). Isolation of glia from Alzheimer’s mice reveals inflammation and dysfunction. Neurobiol. Aging.

[47] Hao Y (2021). Integrated analysis of multimodal single-cell data. Cell.

[48] RStudio Team. *RStudio: Integrated Development Environment for R* (RStudio, 2021).

[49] Korsunsky I (2019). Fast, sensitive and accurate integration of single-cell data with Harmony. Nat. Methods.

[50] Xie Z (2021). Gene set knowledge discovery with Enrichr. Curr. Protoc..

[51] Kolde, R. Pheatmap: pretty heatmaps. https://CRAN.R-project.org/package=pheatmap/index.html (2019).

[52] Hafemeister C, Satija R (2019). Normalization and variance stabilization of single-cell RNA-seq data using regularized negative binomial regression. Genome Biol..

[53] Xu N (2017). Fast free-of-acrylamide clearing tissue (FACT)—an optimized new protocol for rapid, high-resolution imaging of three-dimensional brain tissue. Sci. Rep..

[54] Chiu C-L, Clack N (2022). napari: a Python multi-dimensional image viewer platform for the research community. Microsc. Microanal..

[55] Lunde LK (2015). Postnatal development of the molecular complex underlying astrocyte polarization. Brain Struct. Funct..

[56] Prydz A (2017). Subcellular expression of aquaporin-4 in substantia nigra of normal and MPTP-treated mice. Neuroscience.

[57] Fiala JC (2005). Reconstruct: a free editor for serial section microscopy. J. Microsc..

