# Supplementary material for: Distinct origin and region-dependent contribution of stromal fibroblasts to fibrosis following traumatic injury in mice
Source: Nat Neurosci. 2024 Jun 7;27(7):1285–98. doi: 10.1038/s41593-024-01678-4 (PMC11239523; doi:10.1038/s41593-024-01678-4)
Supplement: Supplementary file 1 — Supplementary Figs. 1–11. [file 41593_2024_1678_MOESM1_ESM.pdf]

nature neuroscience

Supplementary information https://doi.org/10.1038/s41593-024-01678-4

Distinct origin and region-dependent contribution of stromal fibroblasts to fibrosis following traumatic injury in mice

In the format provided by the authors and unedited

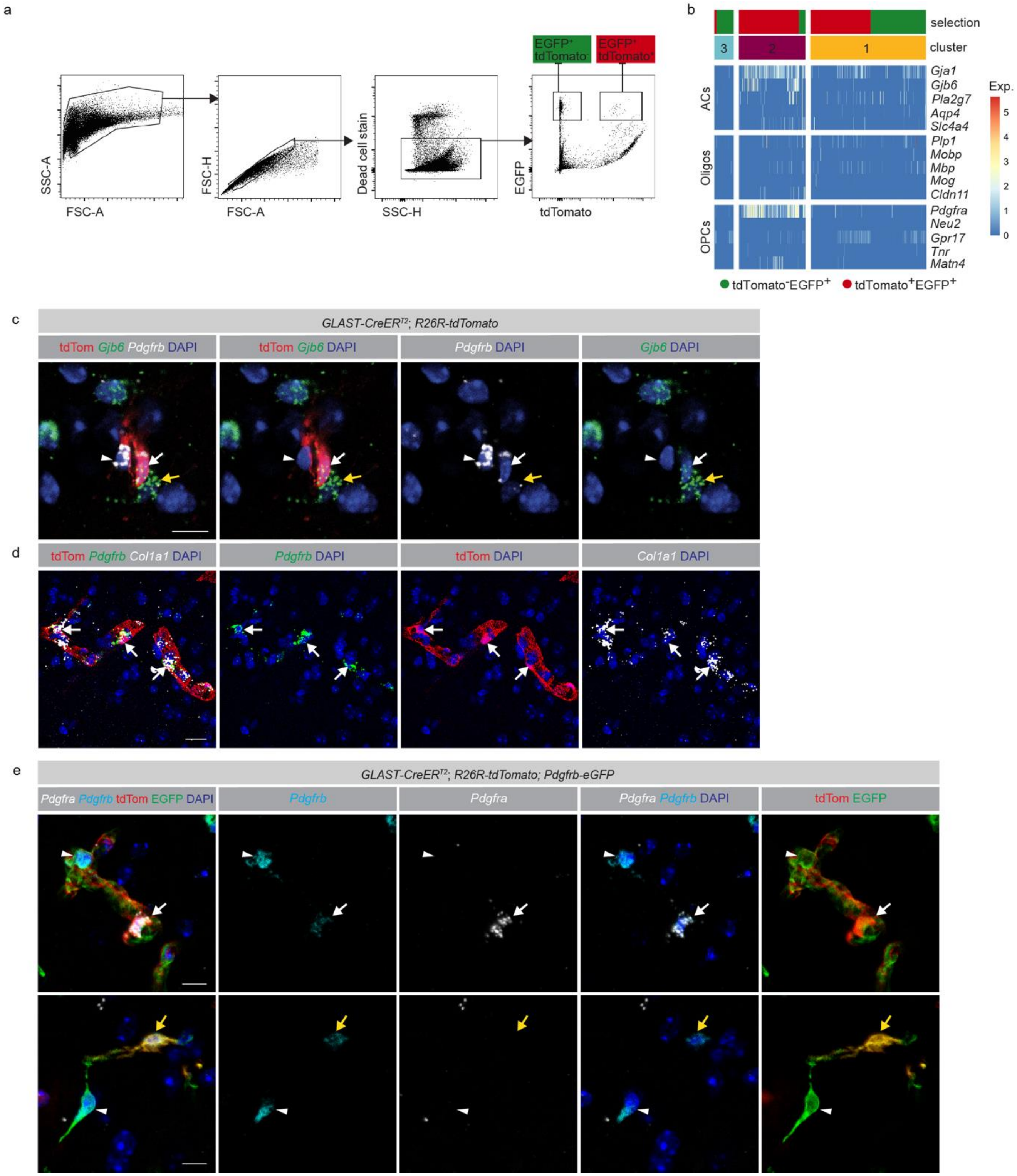

Supplementary Data Figure 1 | **A subset of GLAST^{+} perivascular cells expresses fibroblast markers.**

(**a**) Gating strategy for fluorescence-activated cell sorting of tdTomato^{+}EGFP^{+} and tdTomato^{-}EGFP^{+} perivascular cells for single-cell RNA-sequencing (scRNAseq) (**b**) Heatmap of scRNAseq data of perivascular cells from the uninjured spinal cord of adult *GLAST-CreER^{T2};R26R-tdTomato;Pdgfrb-eGFP* mice separated according to tdTomato^{-}EGFP^{+} (green) and tdTomato^{+}EGFP^{+} (red) cell origin and clusters 1-3, based on Fig. 1d. Gene expression level of marker genes for astrocytes (ACs), oligodendrocytes (Oligos) and oligodendrocyte progenitor cells (OPCs) are shown; scale: log counts.

(**c**) A subset of *GLAST-CreER*^{T2} tdTomato$^+$ perivascular cells co-expresses *Gjb6* and *Pdgfrb* (white arrows) in the uninjured spinal cord. Yellow arrows and white arrowheads point at a tdTomato$^-$*Pdgfrb*$^-$*Gjb6*$^+$ astrocyte and a tdTomato$^-$*Pdgfrb*$^+$*Gjb6*$^-$ mural cell, respectively. (**d**) *GLAST-CreER*^{T2} tdTomato$^+$ perivascular cells on large blood vessels co-express *Pdgfrb* and *Col1a1* (white arrows) in the uninjured spinal cord. (**e**) Upper panel: *Pdgfra* is expressed in a subset of *GLAST-CreER*^{T2} tdTomato$^+$*Pdgfrb*-EGFP$^+$ perivascular cells with low *Pdgfrb* expression (white arrows). In comparison, *Pdgfra* is not expressed by an adjacent tdTomato$^-$EGFP$^+$ perivascular cell with high *Pdgfrb* expression (white arrowheads). Lower panel: *GLAST-CreER*^{T2} tdTomato$^+$*Pdgfrb*-EGFP$^+$ (yellow arrows) and tdTomato$^-$*Pdgfrb*-EGFP$^+$ (white arrowheads) pericytes in small microvessels do not express *Pdgfra* in the uninjured spinal cord. Scale bars: 10 µm (**b**, **d**) and 20 µm (**c**). Cell nuclei are labeled with DAPI. Images are representative of two independent experiments. All images show transverse sections.

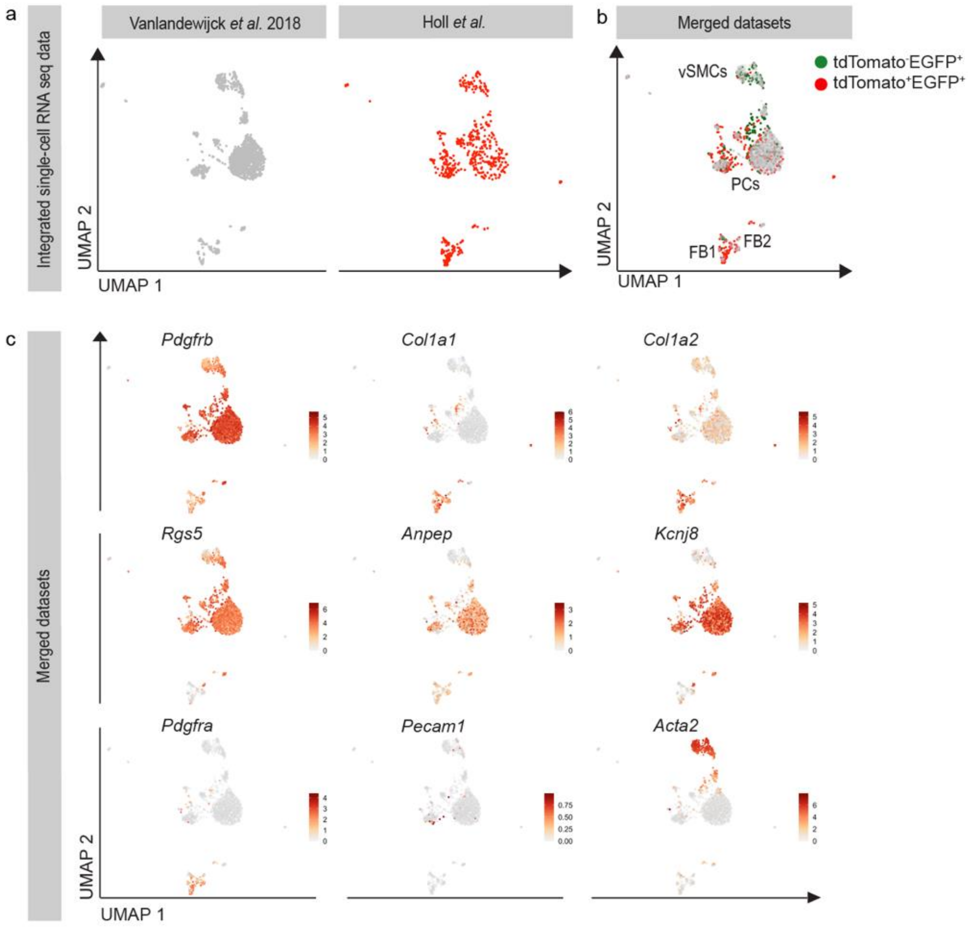

Supplementary Data Figure 2 | **The majority of GLAST^{+} perivascular cells classify transcriptionally as pericytes.**

(**a**) Separate UMAP representations for integrated datasets of uninjured adult brain perivascular cells by Vanlandewijck *et al*. 2018[11] (http://betsholtzlab.org/VascularSingleCells/database.html) and uninjured adult spinal cord perivascular cells as in Fig. 1c-e. (**b**) UMAP representation of merged datasets with color-coding for tdTomato^{+}EGFP^{+} cells (red) and tdTomato^{-}EGFP^{+} cells (green), annotated according to Vanlandewijck *et al*., 2018[11]: FB1 (fibroblasts 1), FB2 (fibroblasts 2), PCs (pericytes), vSMCs (vascular smooth muscle cells). (**c**) Gene expression of selected genes to identify the position of different cell populations within the dimension reduction plot: *Rgs5, Anpep* and *Kcnj8* (pericytes); *Pdgfrb* and *Col1a2* (mural cells/fibroblasts); *Pdgfra* and *Col1a1* (fibroblasts); *Pecam1* (endothelial cells); *Acta2* (vascular smooth muscle cells). Scale: log counts. uninjured adult spinal cord perivascular cells as in Fig. 1c-e. (**b**) UMAP representation of merged datasets with color-coding for tdTomato^{+}EGFP^{+} cells (red) and tdTomato^{-}EGFP^{+} cells (green), annotated according to Vanlandewijck *et al*., 2018[11]: FB1 (fibroblasts 1), FB2 (fibroblasts 2), PCs (pericytes), vSMCs (vascular smooth muscle cells). (**c**) Gene expression of selected genes to identify the position of different cell populations within the dimension reduction plot: *Rgs5, Anpep* and *Kcnj8* (pericytes); *Pdgfrb* and *Col1a2* (mural cells/fibroblasts); *Pdgfra* and *Col1a1* (fibroblasts); *Pecam1* (endothelial cells); *Acta2* (vSMCs). Scale: log counts.

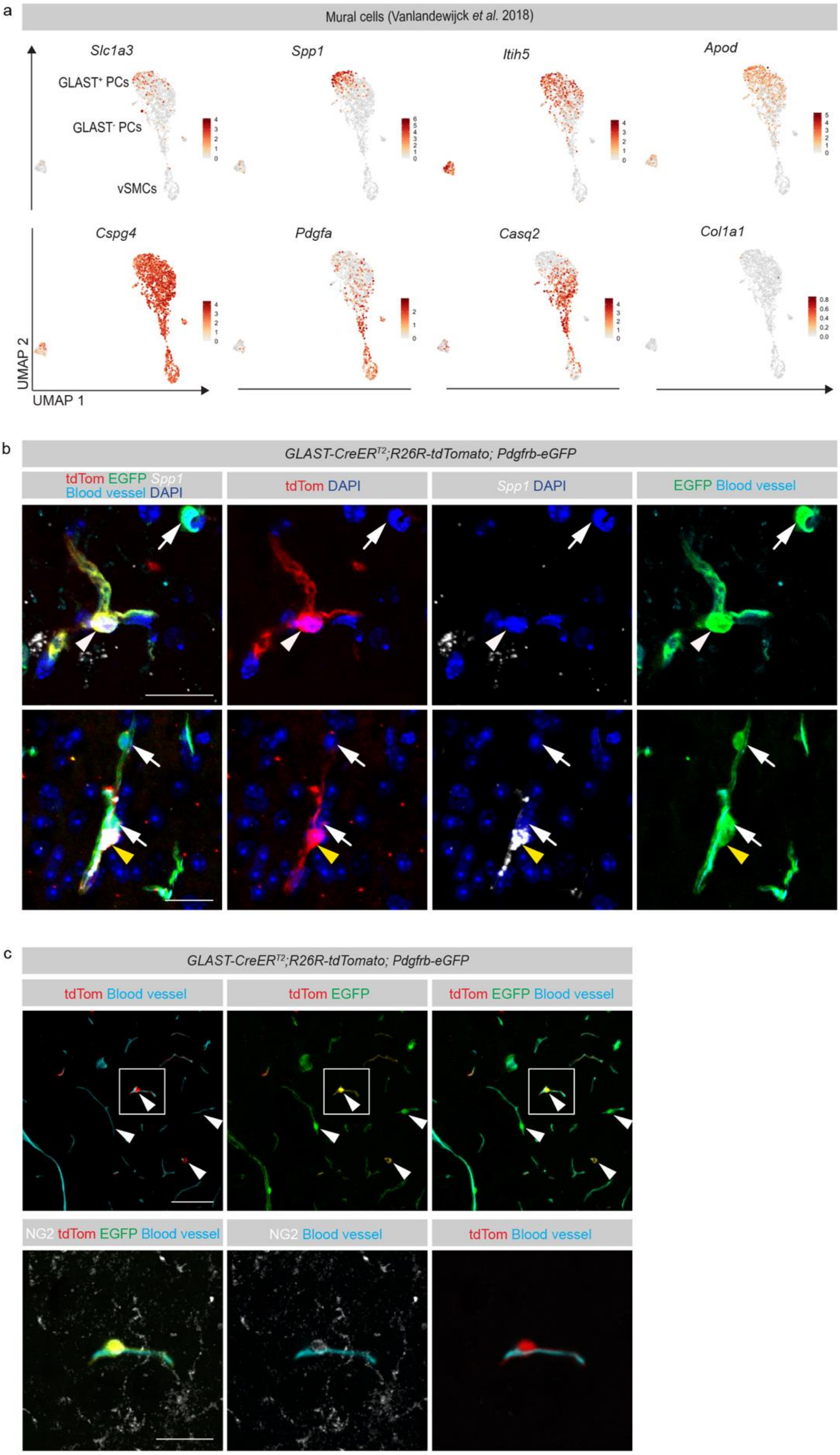
a
Mural cells (Vanlandewijck et al. 2018)
Slc1a3
Spp1
Itih5
Apod
GLAST+ PCs
GLAST- PCs
vSMCs
Cspg4
Pdgfa
Casq2
Col1a1
UMAP 2
UMAP 1
b
GLAST-CreERT2;R26R-tdTomato; Pdgfrb-eGFP
tdTom EGFP Spp1 Blood vessel DAPI
tdTom DAPI
Spp1 DAPI
EGFP Blood vessel
c
GLAST-CreERT2;R26R-tdTomato; Pdgfrb-eGFP
tdTom Blood vessel
tdTom EGFP
tdTom EGFP Blood vessel
NG2 tdTom EGFP Blood vessel
NG2 Blood vessel
tdTom Blood vessel

Supplementary Data Figure 3 | ***Spp1* is expressed in GLAST$^+$ perivascular cells of the arteriole-capillary transitional zone.**

(**a**) Single-cell transcriptomes of mural cells (pericytes (PCs) and vascular smooth muscle cells (vSMCs)) of the adult uninjured brain were obtained from Vanlandewijck *et al.* 2018[11] (http://betsholtzlab.org/VascularSingleCells/database.html) and re-clustered. The resulting UMAP representations display the expression of genes demarcating potential subpopulations within mural cells: *Slc1a3*$^+$ (GLAST$^+$ PCs), *Spp1*$^+$, *Ithih5*$^+$ and *Apod*$^+$ cells separate from *Slc1a3*$^-$ (GLAST$^-$ PCs), *Pdgfa*$^+$ and *Casq2*$^+$ cells and vSMCs. (**b**) In the uninjured spinal cord, *Spp1* is not detected in *GLAST-CreER*^{T2} tdTomato$^+$*Pdgfrb-eGFP*$^+$ pericytes associated with small capillaries (white arrowheads) but is expressed in tdTomato$^+$EGFP$^+$ cells of the post-arteriole transitional zone with projections into the capillary bed (yellow arrowheads), adjacent to tdTomato$^-$EGFP$^+$*Spp1*$^-$ pericytes (white arrows). (**c**) Top row: Transverse view of the uninjured spinal cord grey matter of *GLAST-CreER*^{T2}*;R26R-tdTomato;Pdgfrb-eGFP* mice showing the distribution of tdTomato$^+$EGFP$^+$ and tdTomato$^-$EGFP$^+$ pericytes (white arrowheads) in the capillary bed; Bottom row: closeup of boxed region showing that tdTomato$^+$EGFP$^+$ pericytes express NG2 (chondroitin sulfate proteoglycan 4, gene name *Cspg4*). The blood vessel lumen in (**b**,**c**) was labeled by transcardial perfusion with Alexa Fluor 647-conjugated bovine serum albumin in a gelatin solution. Scale bars: 20 µm (**b**,**c**). Cell nuclei are labeled with DAPI. Images are representative of two independent experiments. All images show transverse sections.

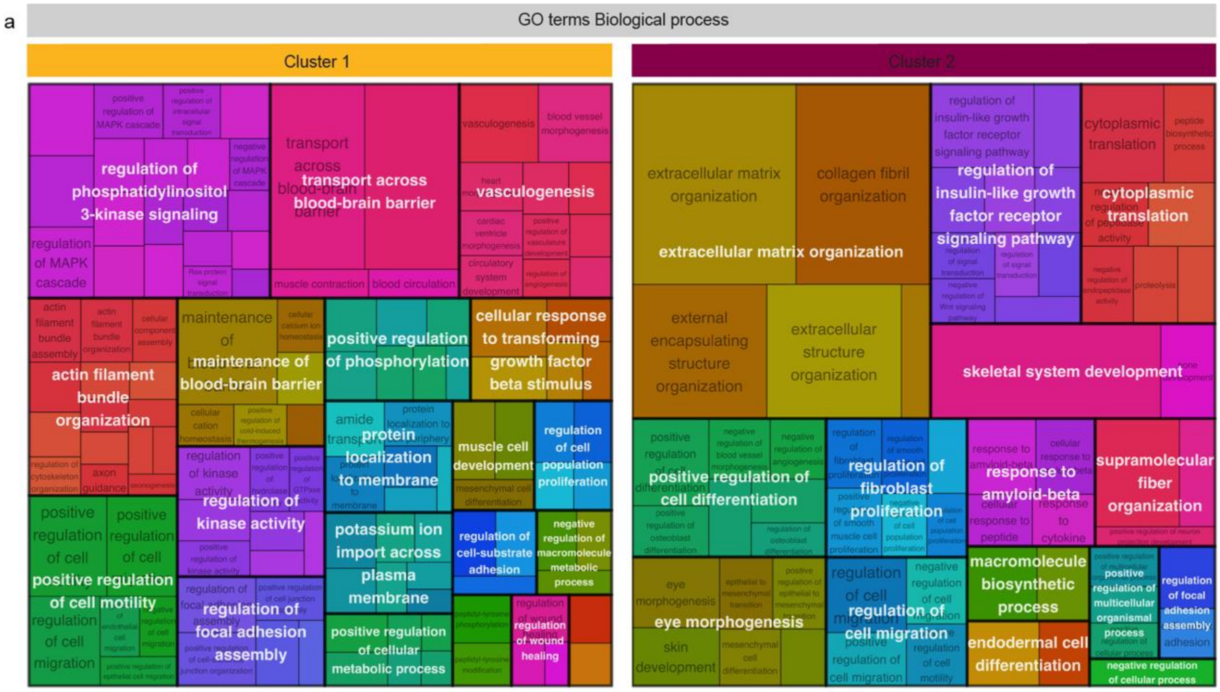

Supplementary Data Figure 4 | **GO term analysis differentiates between GLAST$^+$ pericyte and fibroblast populations.**

(**a**) Biological process gene ontology (GO) enrichment analysis for differentially expressed genes comparing tdTomato$^+$;EGFP$^+$ perivascular cells of clusters 1 and 2 in Figure 1f. Similar GO terms are summarized as global function with Rrvgo package for R.

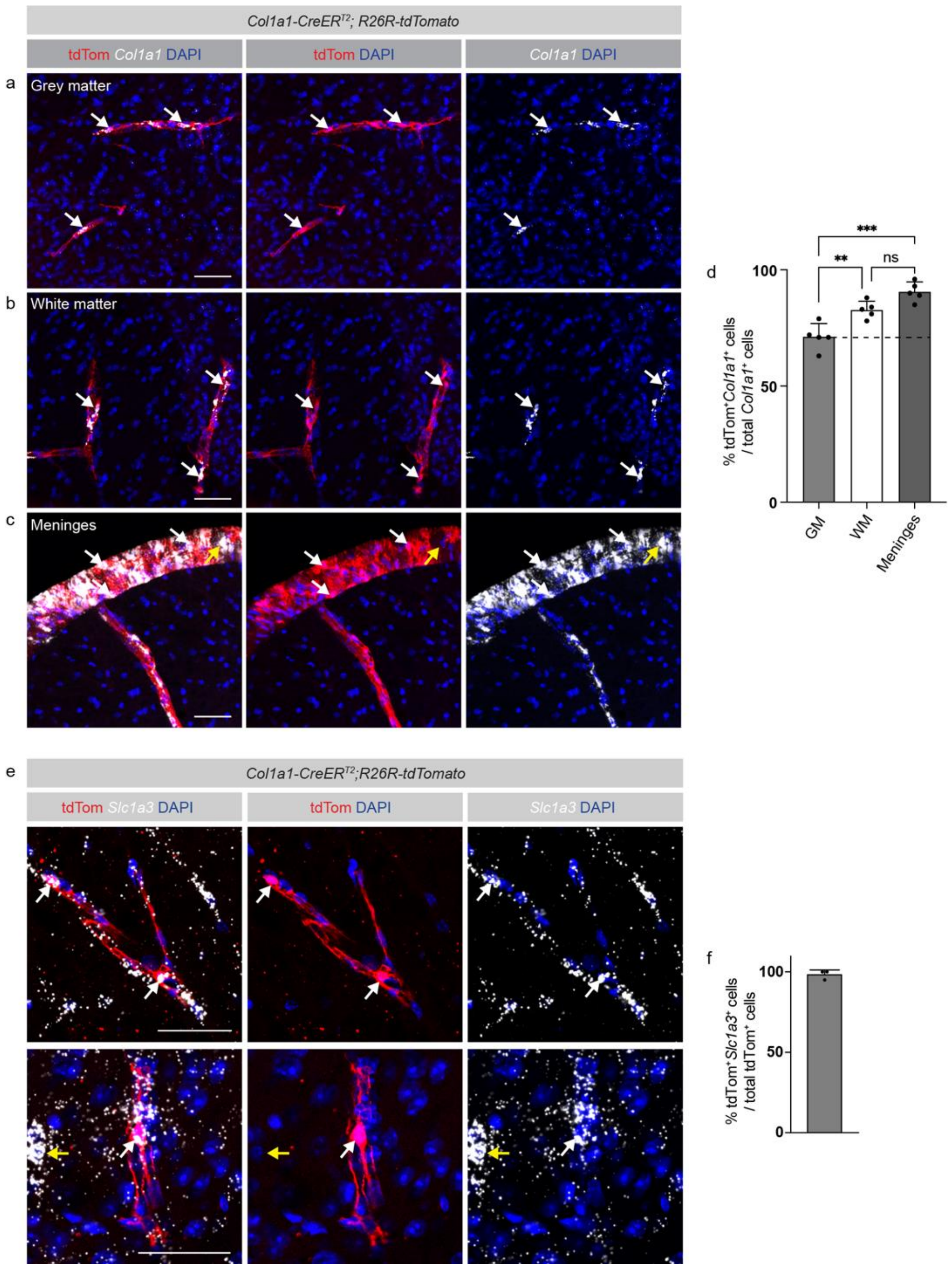

Supplementary Data Figure 5 | ***Col1a1-CreER*^{T2}*;R26R-tdTomato* transgenic mice reflect *Col1a1* mRNA expression.**

(**a-c**) *Col1a1-CreER*^{T2} tdTomato^{+} fibroblasts express *Col1a1* (white arrows) in the uninjured spinal cord (**a**) grey matter, (**b**) white matter and (**c**) meninges. Yellow arrow points to a tdTomato^{-}*Col1a1*$^{+}$ cell. (**d**) Quantification of the recombination efficacy in *Col1a1-CreER*^{T2}*;R26R-tdTomato* mice, determined as the percentage of tdTomato^{+} cells that express *Col1a1* out of total *Col1a1*$^{+}$ cells. *Col1a1* mRNA was detected by RNAscope *in situ* hybridization. Cells with punctate dots in clusters were considered *Col1a1*$^{+}$. Dashed line: mean recombination efficiency in GM = 71.2%. (**e**) *Slc1a3* is widely expressed in astrocytes and virtually all *Col1a1-CreER*^{T2} tdTomato^{+} perivascular fibroblasts (white arrows) throughout the uninjured spinal cord; tdTomato^{-}*Slc1a3*$^{+}$ cells (yellow arrows).

(**f**) Percentage of tdTomato$^+$ cells that express *Slc1a3* out of total tdTomato$^+$ cells. GM, grey matter; WM, white matter. All scale bars: 50 µm. Data shown as mean + SD in (**d**,**f**). n=4-5 (**d**) and n=3 animals (**f**). ns (non-significant)=0.0582, **p=0.0053, ****p<0.0001 by One-Way ANOVA followed by Šídák's multiple comparisons test in (**d**). Cell nuclei are labeled with DAPI. All images show transverse sections. Images are representative of two independent experiments. Source data are provided as a Source Data file.

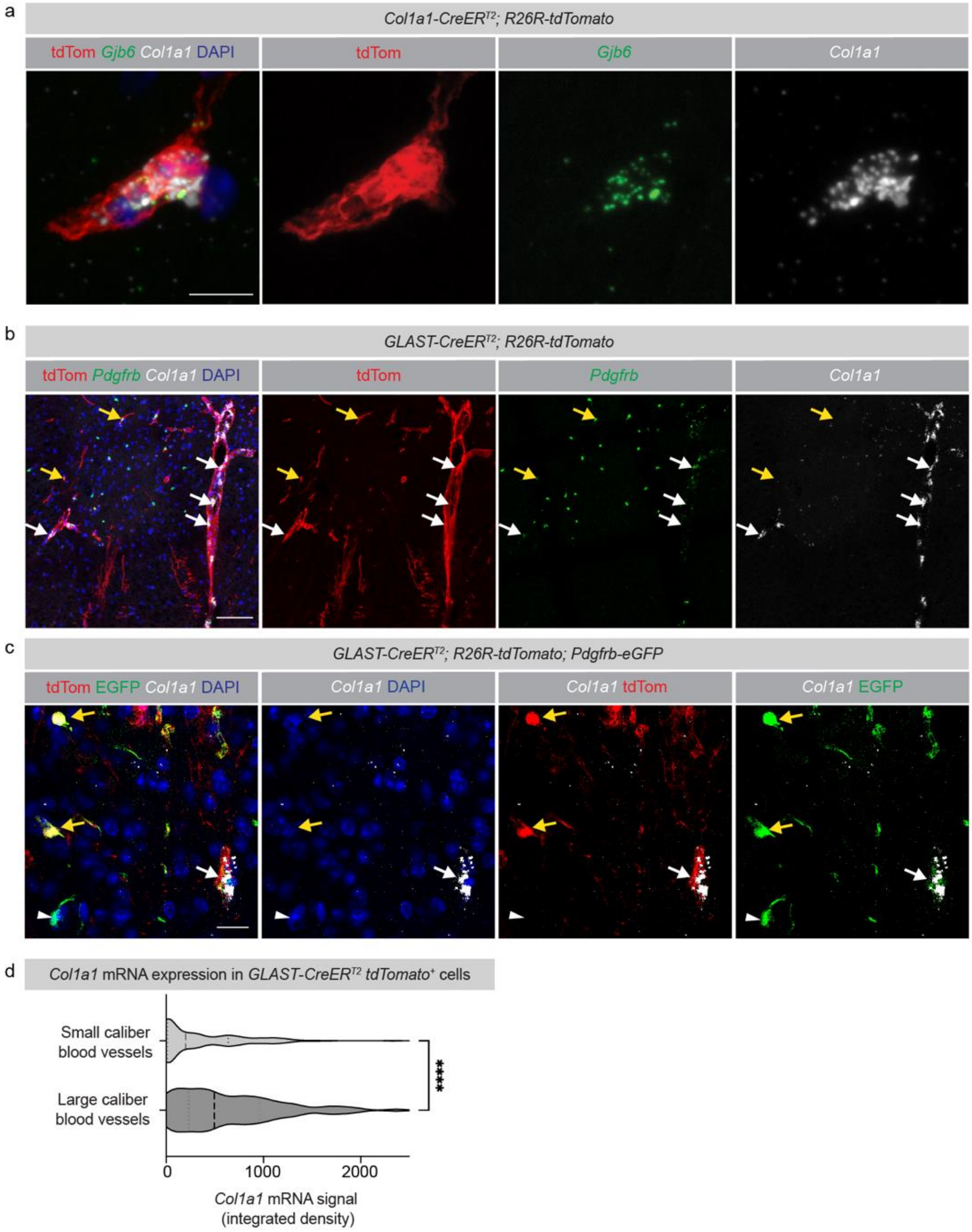

Supplementary Data Figure 6 | **GLAST$^+$ fibroblasts associated with large blood vessels express *Col1a1*, whereas GLAST$^+$ microvascular pericytes do not.**

(**a**) *Gjb6* and *Col1a1* expression in *Col1a1-CreER^{T2}* tdTomato$^+$ fibroblasts in the uninjured spinal cord. (**b**) Robust expression of *Col1a1* in *GLAST-CreER^{T2}* tdTomato$^+$*Pdgfrb*$^+$ perivascular cells along large blood vessels (white arrows) in the uninjured spinal cord. Little to no expression of *Col1a1* is observed in tdTomato$^+$*Pdgfrb*$^+$ cells on small microvessels in the grey matter (yellow arrows). (**c**) *GLAST-CreER^{T2}* tdTomato$^+$*Pdgfrb*-EGFP$^+$ microvascular pericytes with round cell bodies do not express *Col1a1* (yellow arrows), whereas *GLAST-CreER^{T2}* tdTomato$^+$*Pdgfrb*-EGFP$^+$ fibroblasts show high expression of *Col1a1* (white arrow) in the uninjured spinal cord. White arrowheads point to a tdTomato$^-$*Pdgfrb*-EGFP$^+$*Col1a1*$^-$ mural cell.

(**d**) Quantification of *Col1a1* mRNA expression in *GLAST-CreER*^{T2} tdTomato^{+}*Pdgfrb*$^{+}$ perivascular cells associated with small caliber blood vessels, including the arteriole-capillary transitional zone (n=187 cells; mean integrated density: 355.9, median: 494) and large caliber blood vessels (n=97 cells; mean integrated density: 634.6, median: 201.3) in the uninjured spinal cord. Scale bars: 10 µm (**a**), 100 µm (**b**) and 20 µm (**c**). ****p<0.0001 by two-sided, unpaired Student's t test in (**d**). Cell nuclei are labelled with DAPI. All images show transverse sections. Images are representative of two independent experiments. Source data is provided as a Source Data file.

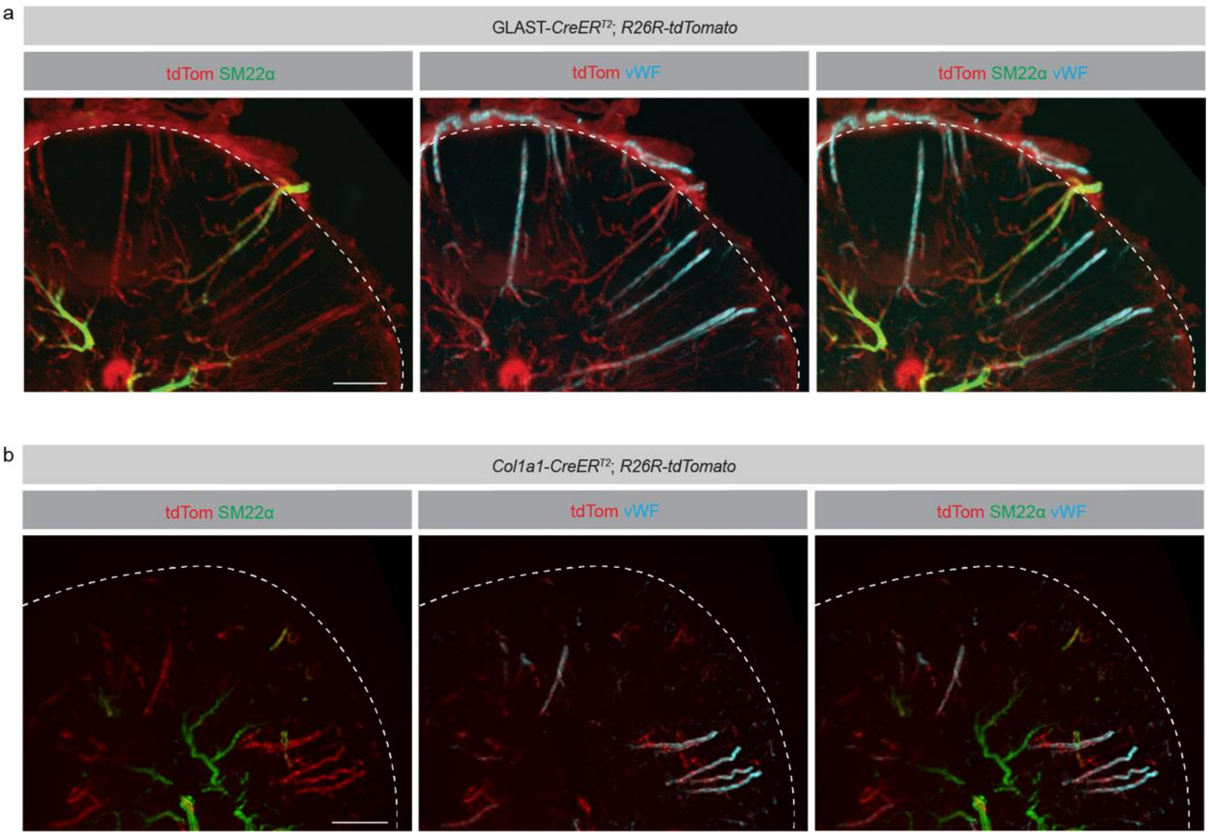

Supplementary Data Figure 7 | **Distribution of GLAST⁺ and *Col1a1*⁺ perivascular cells along spinal cord arterioles and venules.**

(**a**,**b**) Volumetric imaging of 500 μm thick uninjured spinal cord thoracic segments of (**a**) *GLAST-CreER^{T2};R26R-tdTomato* and (**b**) *Col1a1-CreER^{T2};R26R-tdTtomato* mice, co-stained with antibodies against SM22α (arteries/arterioles) and vWF (veins/venules); dashed lines outline the meningeal surface. All scale bars: 200 μm. Images are representative of two independent experiments. All images show transverse sections.

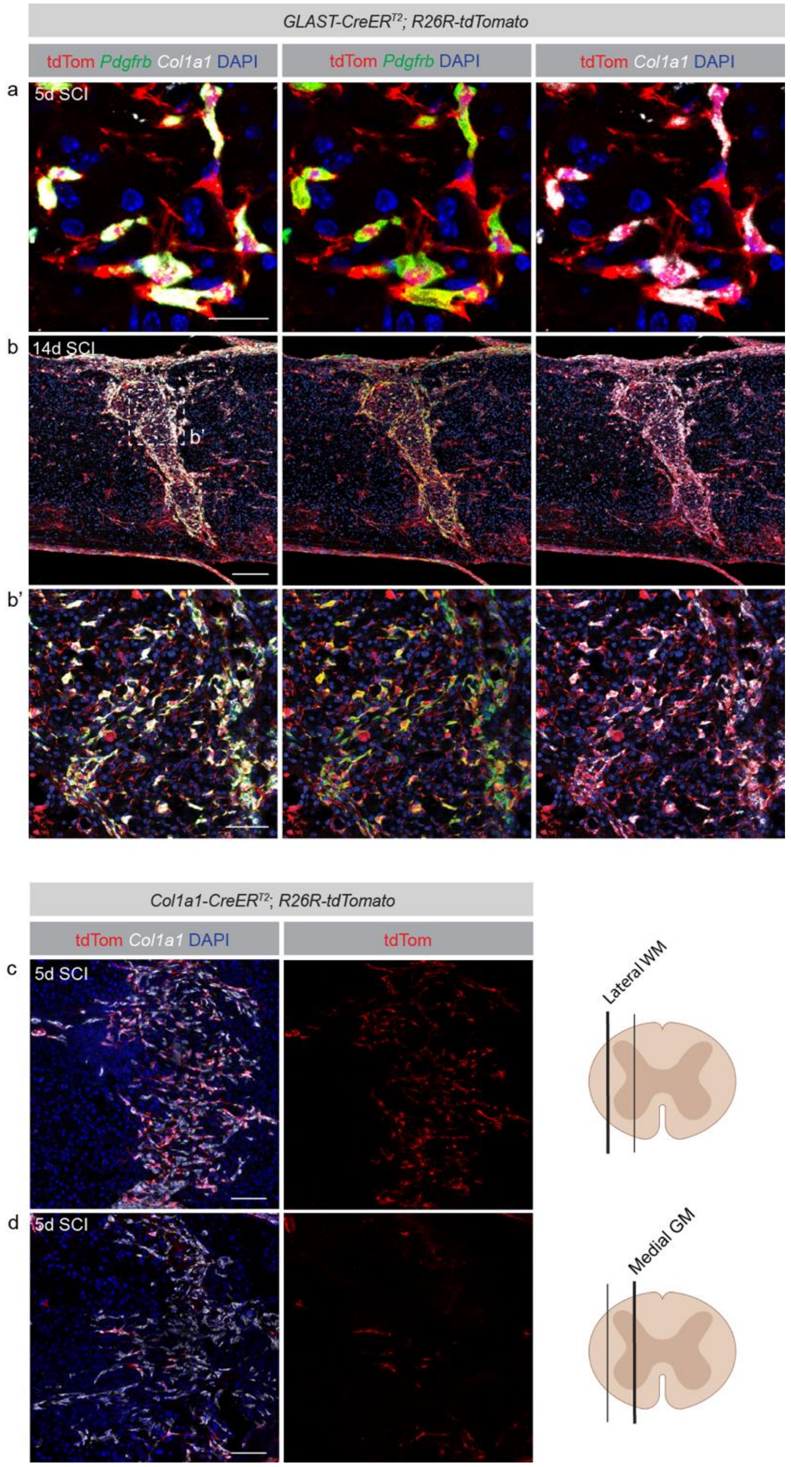

Supplementary Data Figure 8 | **Scar-forming fibroblasts express *Col1a1* but only a fraction derives from *Col1a1*-expressing fibroblasts.**

(**a**,**b**) Lineage tracing using *GLAST-CreER^{T2};R26R-tdTomato* mice reveals that virtually all *Col1a1*$^{+}$*Pdgfrb*$^{+}$ scar-forming fibroblasts are derived from GLAST-expressing perivascular cells (tdTomato^{+}) at (**a**) 5 days and (**b**) 14 days after complete spinal cord crush. (**b'**) shows close-ups of boxed area in (b). (**c**,**d**) Lineage tracing using *Col1a1-CreER^{T2};R26R-tdTomato* mice shows that (**c**) numerous *Col1a1*$^{+}$ scar-forming fibroblasts are derived from *Col1a1*-expressing fibroblasts (tdTomato^{+}) in lateral spinal cord regions (mostly white matter), whereas (**d**) tdTomato^{+} fibroblasts contribute less extensively to *Col1a1*$^{+}$ scar-forming fibroblasts in medial regions (mostly grey matter) of the lesion, at 5 days after complete spinal cord crush.

Scale bars: 20 μm (**a**), 200 μm (**b**), 50 μm (**b′**) and 100 μm (**c**,**d**). Cell nuclei are labelled with DAPI. Images are representative of two independent experiments. All images show sagittal sections. Illustrations were created with BioRender.com.

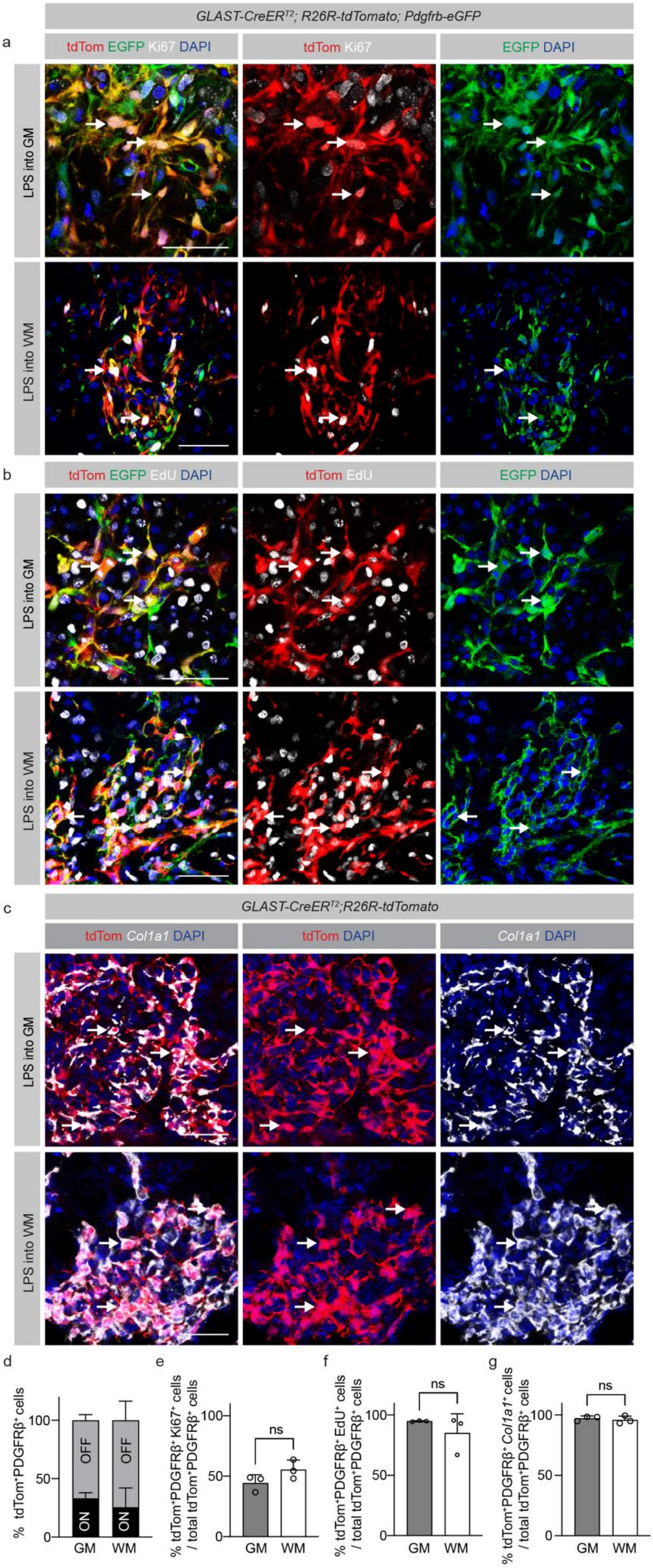

GLAST-CreER^{T2}; R26R-tdTomato; Pdgfrb-eGFP
a
tdTom EGFP Ki67 DAPI
tdTom Ki67
EGFP DAPI
LPS into GM
LPS into WM
b
tdTom EGFP EdU DAPI
tdTom EdU
EGFP DAPI
LPS into GM
LPS into WM
c
GLAST-CreER^{T2};R26R-tdTomato
tdTom Col1a1 DAPI
tdTom DAPI
Col1a1 DAPI
LPS into GM
LPS into WM
d
% tdTom^{+}PDGFRβ^{+} cells
OFF
ON
GM
WM
e
% tdTom^{+}PDGFRβ^{+} Ki67^{+} cells / total tdTom^{+}PDGFRβ^{+} cells
ns
f
% tdTom^{+}PDGFRβ^{+} EdU^{+} cells / total tdTom^{+}PDGFRβ^{+} cells
ns
g
% tdTom^{+}PDGFRβ^{+} Col1a1^{+} cells / total tdTom^{+}PDGFRβ^{+} cells
ns

Supplementary Data Figure 9 | **GLAST^{+} pericytes and fibroblasts in grey and white matter regions react similarly to local LPS-induced inflammation.**

(**a-c**) Comparison of the cellular reaction at 5 days after focal injection of LPS in the grey (GM, upper row) and white matter (WM, lower row) of *GLAST-CreER^{T2}*;*R26R-tdTomato*;*Pdgfrb-eGFP* mice. TdTomato^{+}EGFP^{+} cells proliferate (white arrows) after LPS injection in both grey and white matter, as assessed by (**a**) Ki67 and (**b**) EdU labeling. EdU incorporation reflects the cells that proliferated between day 0 and 5 after intraspinal injection of LPS. (**c**) Nearly all *GLAST-CreER^{T2}* tdTomato^{+} cells express *Col1a1* after LPS injection in both grey and white matter. White arrows point to tdTomato^{+}*Col1a1*$^{+}$ cells. (**d**) Percentage of *GLAST-CreER^{T2}* tdTomato^{+} cells that express PDGFRβ (tdTom^{+}PDGFRβ^{+} cells) associated with (ON vessel) or located outside (OFF vessel) the vascular wall. (**e**) Percentage of proliferating (Ki67^{+}) tdTom^{+}PDGFRβ^{+} cells out of total tdTom^{+}PDGFRβ^{+} cells. (**f**) Percentage of proliferating (EdU^{+}) tdTom^{+}PDGFRβ^{+} cells out of total tdTom^{+}PDGFRβ^{+} cells. (**g**) Percentage of tdTom^{+}PDGFRβ^{+} cells that express *Col1a1* out of total tdTom^{+}PDGFRβ^{+} cells. None of the comparisons in (**e-g**) showed significant differences between grey and white matter inflammation sites. Data shown as mean + SD. N=4 (GM), n=3 (WM) in (d), n=3 (GM and WM) in (e,f,g). ns (non-significant) p= 0.1437 (**e**), p=0.3453 (**f**), p=0.5638 (**g**) by two-sided, unpaired Student's t-test. All scale bars: 50 µm. Images are representative of two independent experiments. Cell nuclei are labelled with DAPI. All images show transverse sections. Source data is provided as a Source Data file.

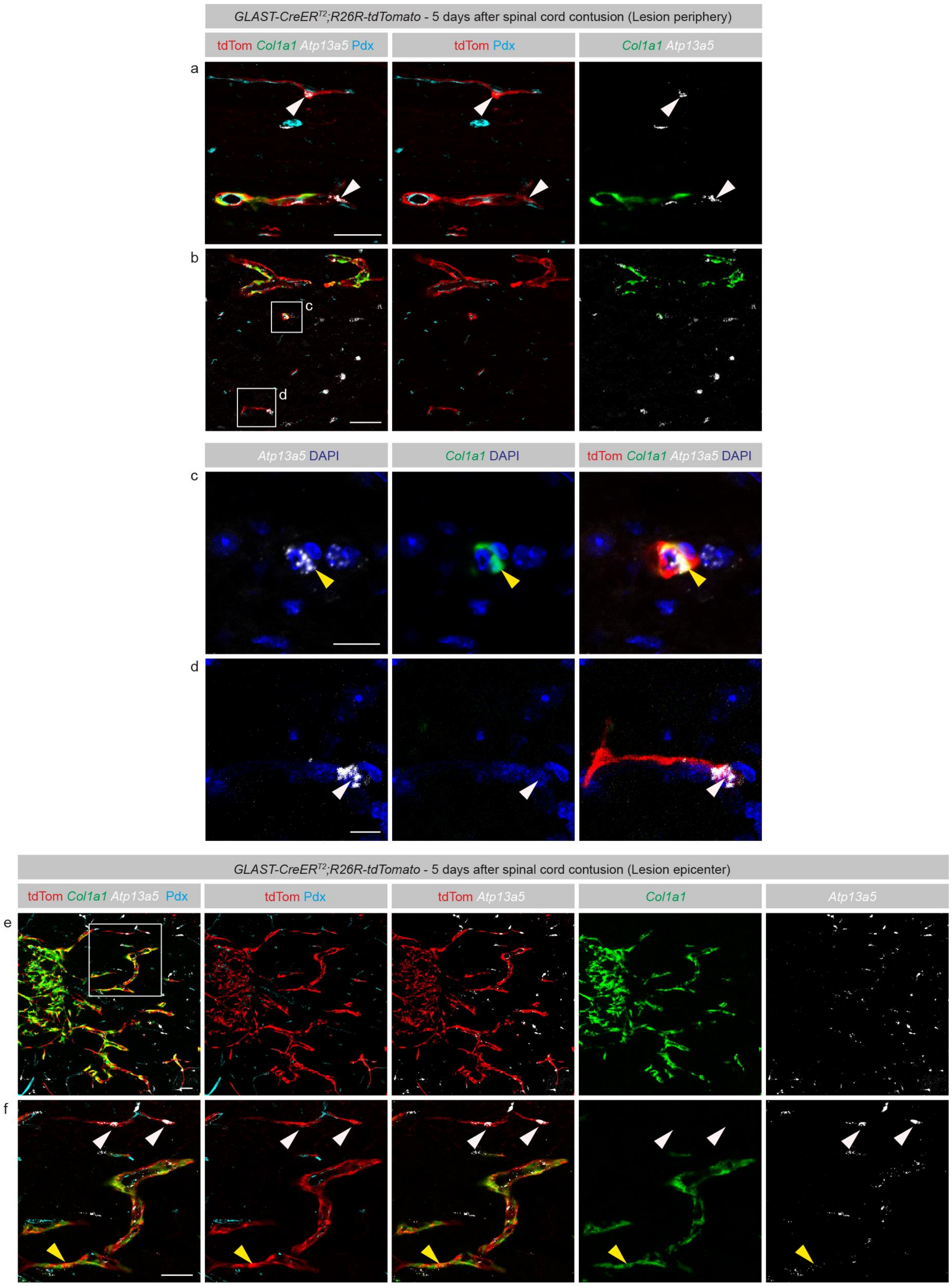
GLAST-CreER^{T2};R26R-tdTomato - 5 days after spinal cord contusion (Lesion periphery)
tdTom Col1a1 Atp13a5 Pdx
tdTom Pdx
Col1a1 Atp13a5
a
b
c
d
Atp13a5 DAPI
Col1a1 DAPI
tdTom Col1a1 Atp13a5 DAPI
c
d
GLAST-CreER^{T2};R26R-tdTomato - 5 days after spinal cord contusion (Lesion epicenter)
tdTom Col1a1 Atp13a5 Pdx
tdTom Pdx
tdTom Atp13a5
Col1a1
Atp13a5
e
f

Supplementary Data Figure 10 | **Expression of the pericyte marker *Atp13a5* rapidly decreases while *Col1a1* expression increases during the transition from pericytes to fibroblasts.**

(**a-f**) Detection of *Col1a1* and *Atp13a5* in the spinal cord of *GLAST-CreER^{T2};R26R-tdTomato* mice, at 5 days after spinal contusion. (**a**,**b**) *Atp13a5* is expressed by tdTomato^{+} (white arrowheads) and tdTomato^{-} pericytes, whereas *Col1a1* is expressed by tdTomato^{+} cells mostly around large blood vessels (podocalyxin^{+}, Pdx) in the lesion periphery and uninjured tissue. (**c**,**d**) Magnified boxed regions in (b) showing (**c**) a tdTomato^{+}*Atp13a5*$^{+}$ pericyte (yellow arrow) surrounding a smaller blood vessel simultaneously expressing *Col1a1* and (**d**) a tdTomato^{+}*Atp13a5*$^{+}$*Col1a1*$^{-}$ pericyte (white arrow). (**e**) *Atp13a5*$^{+}$ pericytes are located in the penumbra of the lesion, while nearly all tdTomato^{+}cells in the lesion epicenter express *Col1a1.* (**f**) Magnified boxed region of the lesion penumbra in (e) showing tdTomato^{+}*Atp13a5*$^{+}$ pericytes (white arrowheads) and a blood vessel growing towards the lesion epicenter with tdTomato^{+}*Col1a1*$^{+}$ perivascular cells expressing a small number of *Atp13a5* mRNA transcripts (yellow arrowhead), presumably activated pericytes. Scale bars: 50 µm (**a**,**b**,**e**,**f**) and 10 µm (**c**,**d**). Cell nuclei are labelled with DAPI. Images are representative of two independent experiments. All images show transverse sections.

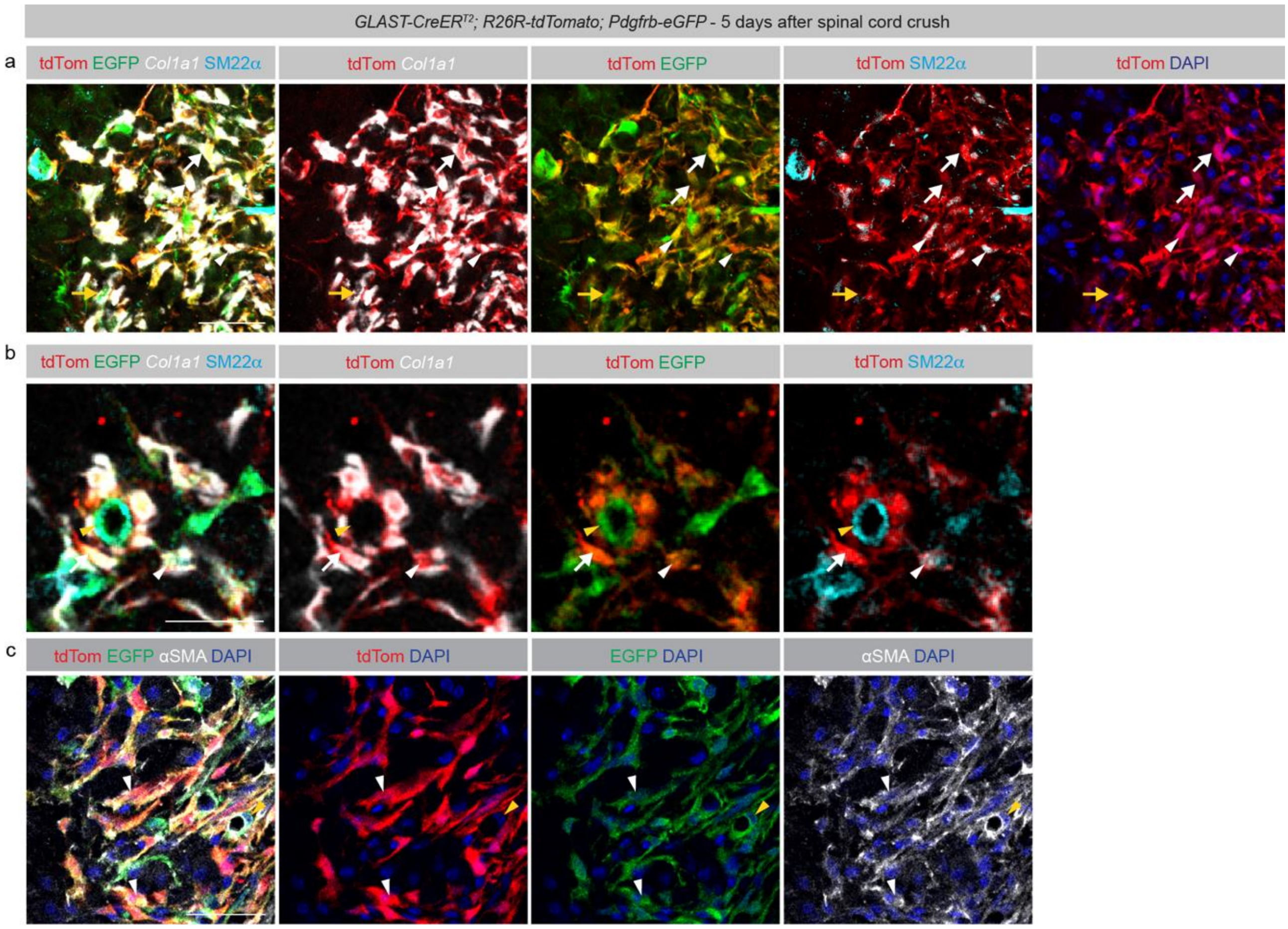

Supplementary Data Figure 11 | **Activated pericytes, fibroblasts and myofibroblasts populate the lesion site after SCI**

(**a-c**) Sagittal views of the spinal cord of *GLAST-CreER^{T2};R26R-tdTomato;Pdgfrb-eGFP* mice at 5 days after complete spinal cord crush. (**a**, **b**) RNAscope *in situ* hybridization for detection of *Col1a1* mRNA in combination with smooth muscle protein 22-alpha (SM22a) immunolabeling allow the identification of different scar-forming stromal cell subtypes: activated fibroblasts, described as tdTomato^{+}EGFP^{+}*Col1a1*$^{+}$SM22a^{-} cells (white arrows in (**a**) and (**b**)), and tdTomato^{-}EGFP^{+}*Col1a1*$^{+}$SM22a^{-} cells (yellow arrow in (**a**)), presumably activated pericytes and/or activated fibroblasts. Myofibroblasts were identified as tdTomato^{+}EGFP^{+}*Col1a1*$^{+}$SM22a^{+} cells (white arrowheads in (**a**) and (**b**)) and vSMCs described as tdTomato^{-}EGFP^{+}*Col1a1*$^{-}$SM22a^{+} cells surrounding blood vessels (yellow arrowhead in (**b**)). (**c**) The expression pattern of aSMA (alpha-smooth muscle actin, gene name *Acta2*) in the scar strongly resembles SM22a expression. Yellow and white arrowheads point to a tdTomato^{-}EGFP^{+}aSMA^{+} mural cell and tdTomato^{+}EGFP^{+}aSMA^{+} myofibroblasts, respectively. All scale bars: 50 µm. Cell nuclei are labeled with DAPI. Images are representative of two independent experiments.
