# Supplementary material for: Distinct origin and region-dependent contribution of stromal fibroblasts to fibrosis following traumatic injury in mice
Source: Nat Neurosci. 2024 Jun 7;27(7):1285–98. doi: 10.1038/s41593-024-01678-4 (PMC11239523; doi:10.1038/s41593-024-01678-4)
Supplement: Supplementary file 2 — Reporting Summary [file 41593_2024_1678_MOESM2_ESM.pdf]

nature portfolio

Corresponding author(s): Christian Göritz

Last updated by author(s): Apr 25, 2024

Reporting Summary

Nature Portfolio wishes to improve the reproducibility of the work that we publish. This form provides structure for consistency and transparency in reporting. For further information on Nature Portfolio policies, see our Editorial Policies and the Editorial Policy Checklist.

Statistics

For all statistical analyses, confirm that the following items are present in the figure legend, table legend, main text, or Methods section.

n/a	Confirmed	
☐	☒	The exact sample size (*n*) for each experimental group/condition, given as a discrete number and unit of measurement
☐	☒	A statement on whether measurements were taken from distinct samples or whether the same sample was measured repeatedly
☐	☒	The statistical test(s) used AND whether they are one- or two-sided<br>*Only common tests should be described solely by name; describe more complex techniques in the Methods section.*
☐	☒	A description of all covariates tested
☐	☒	A description of any assumptions or corrections, such as tests of normality and adjustment for multiple comparisons
☐	☒	A full description of the statistical parameters including central tendency (e.g. means) or other basic estimates (e.g. regression coefficient) AND variation (e.g. standard deviation) or associated estimates of uncertainty (e.g. confidence intervals)
☐	☒	For null hypothesis testing, the test statistic (e.g. *F*, *t*, *r*) with confidence intervals, effect sizes, degrees of freedom and *P* value noted<br>*Give P values as exact values whenever suitable.*
☒	☐	For Bayesian analysis, information on the choice of priors and Markov chain Monte Carlo settings
☒	☐	For hierarchical and complex designs, identification of the appropriate level for tests and full reporting of outcomes
☒	☐	Estimates of effect sizes (e.g. Cohen's *d*, Pearson's *r*), indicating how they were calculated

Our web collection on statistics for biologists contains articles on many of the points above.

Software and code

Policy information about availability of computer code

Data collection	FACS data/cell sorting was performed on FACSAria III Cell Sorter system (BD Biosciences).<br>Single cells were processed/data was generated according to the Smart-Seq2, Smart-seq 3 and Smart-seq3xpress protocol (Picelli et al., 2014; Hagemann-Jensen, M et al. 2020&2022). Smart-Seq2 samples were sequenced on Illumina HiSeq2500 (HiSeq Control Software 2.2.58/RTA 1.18.64) with a 1x51 setup using 'HiSeq SBS Kit v4' chemistry. Smartseq3 and Smartseq3xpress libraries were sequenced on a Illumina NextSeq 500 (Illumina NextSeq Control Software 2.2.0).<br>Immunofluorescence images were acquired with a TCS SP8 X White Light Laser Confocal Microscope (Leica) using LAS X 3.5.7.23225 software<br>Cleared tissue images were aquired with Zeiss Z.1 lightsheet microscope.<br>Electron microscopic images were acquired with a Tecnai 12 electron microscope (FEI) at 80 kV equipped with ITEM FEI version 5.1 software (Olympus Soft Imaging Solutions, Münster, Germany).
Data analysis	Rstudio version 2022.12.0 with R studio version 1.4.1717 and Seurat version 4.3.0.1 were used to analyze single cell mRNA seq data. DEsingle version 1.18.0 was used for differential gene expression analysis.<br>Graphpad Prism, version 10.1.1 was used for statistical analysis.<br>Confocal image analysis, processing, assembly and cell counting were performed with the open source software ImageJ/Fiji version 2.1.0/1.53c.<br>Adobe Illustrator Version 27.03.1 release for Mac was used for assembly of figures.<br>FACS data were analyzed using FlowJo software v10.8.0.

For manuscripts utilizing custom algorithms or software that are central to the research but not yet described in published literature, software must be made available to editors and reviewers. We strongly encourage code deposition in a community repository (e.g. GitHub). See the Nature Portfolio guidelines for submitting code & software for further information.

Data

Policy information about availability of data

All manuscripts must include a data availability statement. This statement should provide the following information, where applicable:

- Accession codes, unique identifiers, or web links for publicly available datasets
- A description of any restrictions on data availability
- For clinical datasets or third party data, please ensure that the statement adheres to our policy

The authors declare that all data supporting the findings of this study are included in this published article (and its supplementary information files). Source Data for Figures 2, 5 and 6, Extended Data Figures 1 and 2, and Supplementary Figures 5, 6 and 9 are provided with the paper. The publicly available dataset GSE98816 was used for comparisons in extended data figure 2 and mouse reference genome GCF_000001635.20/27. ScRNA-sequencing data are deposited in the Gene Expression Omnibus (GEO) database, accession number GSE229916.

Human research participants

Policy information about studies involving human research participants and Sex and Gender in Research.

Reporting on sex and gender	N/A
Population characteristics	N/A
Recruitment	N/A
Ethics oversight	N/A

Note that full information on the approval of the study protocol must also be provided in the manuscript.

Field-specific reporting

Please select the one below that is the best fit for your research. If you are not sure, read the appropriate sections before making your selection.

☒ Life sciences ☐ Behavioural & social sciences ☐ Ecological, evolutionary & environmental sciences

For a reference copy of the document with all sections, see nature.com/documents/nr-reporting-summary-flat.pdf

Life sciences study design

All studies must disclose on these points even when the disclosure is negative.

Sample size	No statistical methods have been used to predetermine sample size. Based on our previous work (Dias D.O.; Kalkitsas J. et al. Pericyte-derived fibrotic scarring is conserved across diverse central nervous system lesions Nat Commun. 2021 Sep 17;12(1):5501.; Dias, D. O. et al. Reducing Pericyte-Derived Scarring Promotes Recovery after Spinal Cord Injury. Cell 173, 153-165.e22 (2018); Göritz, C. et al. A pericyte origin of spinal cord scar tissue. Science (80-.). 333, (2011)), groups of 3 to 4 animals are sufficient for quantitative analyses, but in most cases, more animals were used.
Data exclusions	As pre-established, for contusion and complete crush spinal cord injury experiments, animals undergoing surgery that reached the human endpoint (e.g. loss of 15% of their pre- operative body-weight) were euthanized.
Replication	Replicate measurements were obtained by analyzing 3 or more alternate tissue sections per animal. Three or more biological replicates were used per condition. All attempts at replication were successful.
Randomization	All animals used in experiments involving contusion and crush spinal cord injury for tissue analysis were female mice with approximately similar age, weight and genetic background. For sequencing experiments and all other experiments, both male and female mice of similar age, weight and genetic background were equally distributed across experimental groups.
Blinding	The investigators were blinded to group allocation during data collection and analyses.

Reporting for specific materials, systems and methods

We require information from authors about some types of materials, experimental systems and methods used in many studies. Here, indicate whether each material, system or method listed is relevant to your study. If you are not sure if a list item applies to your research, read the appropriate section before selecting a response.

Materials & experimental systems

n/a	Involved in the study
☐	☒ Antibodies
☒	☐ Eukaryotic cell lines
☒	☐ Palaeontology and archaeology
☐	☒ Animals and other organisms
☒	☐ Clinical data
☒	☐ Dual use research of concern

Methods

n/a	Involved in the study
☒	☐ ChIP-seq
☐	☒ Flow cytometry
☒	☐ MRI-based neuroimaging

Antibodies

Antibodies used

GFP (1:2000, chicken, Aves Labs; GFP-1020; 1:2000, sheep, Bio-Rad, 4745-1051)
PDGFRβ (1:200, rabbit, Abcam, ab32570; 1:100, rat, eBioscience, 14-1402-82)
Podocalyxin (1:200, goat, R&D Systems, AF1556)
Alpha smooth muscle actin (1:200, rabbit, abcam, ab5694)
SM22a (1:200, rabbit, abcam, ab14106)
NG2 Chondroitin Sulfate Proteoglycan (1:200, rabbit, Milliopore, AB5320; no Triton X-100)
Ki67 (1:2000, rat, eBioscience, 14-5698)
RFP (Red fluorescent protein, 1:250, chicken, Novus Biologicals, NBP1-97371; 1:100, rabbit, Rockland, 600-401-379s)
Mac-2 (1:500, rat, Cedarlane Labs, CL8942B)
vWF (von Willebrand factor, 1:100, sheep, Abcam, ab11713)

The following secondary antibody was used for immunogold cytochemistry (electron microscopy): goat anti-rabbit IgG conjugated to 15 nm gold particles (1:20, Abcam, ab105298)

All secondary antibodies used for immunohistochemistry were F(ab')2 fragment affinity-purified antibodies purchased from Jackson Immunoresearch and diluted at 1:500: Alexa Fluor 488 donkey anti- chicken IgY (703-546-155), Cy3 donkey anti-chicken IgY (703-166-155), Alexa Fluor 647 donkey anti-goat IgG (705-606-147), Alexa Fluor 594 donkey anti-goat IgG (705-585-147), Alexa Fluor 680 donkey anti-goat IgG (705-625-147), Alexa Fluor 488 donkey anti-rabbit IgG (711-546-152), Alexa Fluor 647 donkey anti-rabbit IgG (711-606-152), Alexa Fluor 680 donkey anti-rabbit IgG (771-625-152), Alexa Fluor 488 donkey anti-rat IgG (712-546-153), Alexa Fluor 647 donkey anti-rat IgG (712-606-153), Alexa Fluor 488 donkey anti-sheep IgG (713-546-147), Biotinylated secondary antibodies were revealed with Alexa Fluor 594 conjugated Streptavidin (1:500, Jackson Immunoresearch, 016-580-084) or Alexa Fluor 680 conjugated Streptavidin (1:500, Jackson Immunoresearch, 016-620-084).

Validation

Only previously published antibodies or antibodies with company based validations were used for immunohistochemistry:
PDGFRβ (1:200, rabbit, Abcam, ab32570, clone Y92): Göritz, C. et al. A pericyte origin of spinal cord scar tissue. Science (80-.). 333, (2011); Dias, D. O. et al. Reducing Pericyte-Derived Scarring Promotes Recovery after Spinal Cord Injury. Cell 173, 153-165.e22 (2018); Dias DO, Kalkitsas J et al. Pericyte-derived fibrotic scarring is conserved across diverse central nervous system lesions. Nat Commun. 2021 Sep 17;12(1):5501.

PDGFRβ (1:100, rat, eBioscience, 14-1402-82, clone APB5): Vanlandewijck, M. et al. A molecular atlas of cell types and zonation in the brain vasculature. Nature 554, 475–480 (2018); Diéguez-Hurtado, R. et al. Loss of the transcription factor RBPJ induces disease-promoting properties in brain pericytes. Nat. Commun. 10, 2817 (2019); Dias DO, Kalkitsas J et al. Pericyte-derived fibrotic scarring is conserved across diverse central nervous system lesions. Nat Commun. 2021 Sep 17;12(1):5501.

Podocalyxin (1:200, goat, R&D Systems, AF1556): Dias, D. O. et al. Reducing Pericyte-Derived Scarring Promotes Recovery after Spinal Cord Injury. Cell 173, 153-165.e22 (2018); Vanlandewijck, M. et al. A molecular atlas of cell types and zonation in the brain vasculature. Nature 554, 475–480 (2018); Diéguez-Hurtado, R. et al. Loss of the transcription factor RBPJ induces disease- promoting properties in brain pericytes. Nat. Commun. 10, 2817 (2019); Dias DO, Kalkitsas J et al. Pericyte-derived fibrotic scarring is conserved across diverse central nervous system lesions. Nat Commun. 2021 Sep 17;12(1):5501; https://www.rndsystems.com/products/mouse- podocalyxin-antibody_af1556#product-details

GFP (1:10000, chicken, Aves Labs, GFP-1020): Dias, D. O. et al. Reducing Pericyte-Derived Scarring Promotes Recovery after Spinal Cord Injury. Cell 173, 153-165.e22 (2018); Dias DO, Kalkitsas J et al. Pericyte-derived fibrotic scarring is conserved across diverse central nervous system lesions. Nat Commun. 2021 Sep 17;12(1):5501; https://www.aveslabs.com/products/anti-green-fluorescent-protein-antibody- gfp

GFP (1:500, sheep, Bio-Rad, 4745-1051): Haberlandt, C. et al. Gray Matter NG2 Cells Display Multiple Ca2+-Signaling Pathways and Highly Motile Processes. PLoS One 6, e17575 (2011); Dias DO, Kalkitsas J et al. Pericyte-derived fibrotic scarring is conserved across diverse central nervous system lesions. Nat Commun. 2021 Sep 17;12(1):5501; https://www.bio-rad-antibodies.com/polyclonal/green-fluorescent- protein-antibody-4745-1051.html?f=purified

Red Fluorescent Protein (1:500, rabbit, Rockland, 600-401-379): Anderson, M. A. et al. Required growth facilitators propel axon regeneration across complete spinal cord injury. Nature 561, 396–400 (2018); Dias DO, Kalkitsas J et al. Pericyte-derived fibrotic scarring is conserved across diverse central nervous system lesions. Nat Commun. 2021 Sep 17;12(1):5501; https://rockland-inc.com/store/Antibodies-to- GFP-and-Antibodies-to-RFP-600-401-379-O4L_24299.aspx
Red Fluorescent Protein (1:500, chicken, Novus Biologicals, NBP1-97371): Anderson, M. A. et al. Required growth facilitators propel

axon regeneration across complete spinal cord injury. Nature 561, 396–400 (2018); Dias DO, Kalkitsas J et al. Pericyte-derived fibrotic scarring is conserved across diverse central nervous system lesions. Nat Commun. 2021 Sep 17;12(1):5501; https://www.novusbio.com/products/ rfp-antibody_nbp1-97371

NG2 Chondroitin Sulfate Proteoglycan (1:200, rabbit, Milliopore, AB5320; no Triton X-100): Teichert, M., Milde, L., Holm, A. et al. Pericyte-expressed Tie2 controls angiogenesis and vessel maturation. Nat Commun 8, 16106 (2017). https://doi.org/10.1038/ncomms16106

Ki67 (1:2000, rat, eBioscience, 14-5698): The manufacturer states that advanced validation has been performed by cell treatment and knockout and immunohistochemistry has been confirmed on various tissues and cell liens. Magnusson, J. P. J. P. et al. A latent neurogenic program in astrocytes regulated by Notch signaling in the mouse. Science (80-.); Dias DO, Kalkitsas J et al. Pericyte-derived fibrotic scarring is conserved across diverse central nervous system lesions. Nat Commun. 2021 Sep 17;12(1):5501. 346, 237–41 (2014); https://www.thermofisher.com/antibody/ product/Ki-67-Antibody-clone-SolA15-Monoclonal/14-5698-82

αSMA (1:100, rabbit, Abcam, ab5694): Assinck, P. et al. Myelinogenic plasticity of oligodendrocyte precursor cells following spinal cord contusion injury. J. Neurosci. 37, 8635–8654 (2017); Guimarães-Camboa, N. et al. Pericytes of Multiple Organs Do Not Behave as Mesenchymal Stem Cells In Vivo. Cell Stem Cell 20, 345-359.e5 (2017); Dias DO, Kalkitsas J et al. Pericyte-derived fibrotic scarring is conserved across diverse central nervous system lesions. Nat Commun. 2021 Sep 17;12(1):5501.
SM22a (1:200, rabbit, abcam, ab14106): Vanlandewijck M et al. A molecular atlas of cell types and zonation in the brain vasculature. Nature 554:475-480 (2018); Diéguez-Hurtado R et al. Loss of the transcription factor RBPJ induces disease-promoting properties in brain pericytes. Nat Commun 10:2817 (2019); Eduardo Linck Guimaraes et al. ,Corpora cavernosa fibroblasts mediate penile erection.Science383,eade8064(2024).DOI:10.1126/science.ade8064

Mac-2 (1:500, rat, Cedarlane Labs, CL8942B): Zhan L et al. A MAC2-positive progenitor-like microglial population is resistant to CSF1R inhibition in adult mouse brain. Elife. 2020 Oct 15;9:e51796.

vWF (von Willebrand factor, 1:100, sheep, Abcam, ab11713): Galstyan, A., Markman, J.L., Shatalova, E.S. et al. Blood–brain barrier permeable nano immunoconjugates induce local immune responses for glioma therapy. Nat Commun 10, 3850 (2019). https://doi.org/10.1038/s41467-019-11719-3

Control tissue sections were stained with secondary antibody alone.

Animals and other research organisms

Policy information about studies involving animals; ARRIVE guidelines recommended for reporting animal research, and Sex and Gender in Research

Laboratory animals	The following adult (8 weeks of age or older) female and male mice were used in this study (or combinations of these lines):<br>- GLAST-CreERT2 transgenic mice (Slezak, M. et al. Transgenic mice for conditional gene manipulation in astroglial cells. Glia 55, 1565-1576, doi:10.1002/glia.20570 (2007)) in a C57Bl/6J genetic background.<br>- Rosa26-tdTomato Cre-reporter mice (obtained from the Jackson Laboratory, B6.Cg-Gt(Rosa)26Sortm14(CAG-tdTomato)Hze/J, JAX stock: 007914) in a C57Bl/6J genetic background.<br>- Col1a1-CreERT2 transgenic mice (PHENOMIN-Institut Clinique de la Souris, deposited in the Infrafrontier repository (https://www.infrafrontier.eu/) under the identifier EM:14947) in a C57Bl/6J genetic background<br>- Pdgfrb-eGFP transgenic mice (Tg(Pdgfrb-EGFP)JN169Gsat/Mmucd (RRID:MMRRC_031796-UCD) Gong et al., 2003)<br>- Rasless mice: Drosten, M. et al. Genetic analysis of Ras signalling pathways in cell proliferation, migration and survival. Embo J 29, 1091–1104 (2010).
Wild animals	The study did not involve wild animals.
Reporting on sex	All animals used in experiments involving contusion and crush spinal cord injury for tissue analysis were female mice. For sequencing experiments and all other experiments, both male and female mice of similar age, weight and genetic background were equally distributed across experimental groups. We used mainly female mice for severe injury experiments because they show better recovery after surgery which reduces the risk animals reaching the humane endpoint and finally the total number of animals needed. However, we did not find any differences in contribution to fibrosis when using male mice in earlier experiments.
Field-collected samples	The study did not involve samples collected from the field.
Ethics oversight	All experimental procedures were carried out in accordance with the Swedish and European Union law and guidelines and approved by the regional ethical committees (Stockholm ethical committee/ “Stockholms djurförsöksetiska nämnd”).

Note that full information on the approval of the study protocol must also be provided in the manuscript.

Flow Cytometry

Plots

Confirm that:

☒ The axis labels state the marker and fluorochrome used (e.g. CD4-FITC).

☒ The axis scales are clearly visible. Include numbers along axes only for bottom left plot of group (a 'group' is an analysis of identical markers).

☐ All plots are contour plots with outliers or pseudocolor plots.

☐ A numerical value for number of cells or percentage (with statistics) is provided.

Methodology

Sample preparation	5 days after spinal cord crush injury, animals were euthanized by intraperitoneal injection of sodium pentobarbital (200mg/Kg, 100ul i.p., APL) and transcardially perfused with cold Hank's Balanced Salt Solution without calcium or magnesium (HBSS) (Invitrogen). The vertebral column was immediately removed to cold HBSS, the spinal cord was carefully dissected out of the vertebrae and the crush injury segment was separated from the uninjured spinal cord and treated separately hereafter. After removal of the outer meningeal layers, the tissue was dissociated mechanically with a razor blade on a glass petri dish and immediately transferred to cold HBSS.For SmartSeq2 samples: Followed by enzymatic digestion with papain at a final concentration of 8 U/mL with 80 Kunitz units/mL DNase I (Sigma-Aldrich, D4263) in Ca2+/Mg2+-free piperazine-N,N'-bis(2-ethanesulfonic acid) (PIPES)/cysteine -based buffer, pH 7.4, for 40min at 37°C, 350rpm on a ThermoMixer® (Eppendorf). The tissue was carefully triturated and incubated for additional 5min. The cell suspension was passed through a 70µmCell Strainer (Corning), washed with Minimum Essential Media (MEM) with 1% bovine serum albumin (BSA) and spun down at 200g for 5min at 4°C. The cells were resuspended in MEM and centrifuged over a 90% Percoll gradient (GE Healthcare, 17-0891-01) at 250g for 15min at 4°C. Cells in the lipid layer and below were diluted 5 times in MEM with 1% BSA and spun in a 15mL tube at 250g for 10min at 4°C. All supernatants including the lipid layer were carefully removed and the pellet resuspended in cold MACS Buffer. Prior to Smart-seq 3 and Smart-seq3xpress library preparation, cells were enzymatically digested following manufacturer's instructions from the adult brain dissociation kit. Briefly, cells were incubated with enzymes for 15 min at 37°C with agitation, then triturated by pipetting up and down with 1000 uL tips and incubated for 10 min more. Digestion was stopped by adding 10 mL of MACS buffer (PBS with 0.5% FBS). Cells were filtered through a 70 mm cell strainer and centrifuged 10 min 300g at 4°C.<br>Following the two protocols, pellets were resuspended in cold MACS buffer and magnetic myelin removal beads (Miltenyi Biotech, 130-096-433) and incubated for 15 min at 4°C. The cells were washed and run over MACS MS columns (Miltenyi Biotech, 130-042-201) on a magnetic stand according to the manufacturer's instructions. The cells in the flow through were collected, spun down, resuspended in FACS buffer (2% FBS in PBS) and kept on ice until further processing. For Smart-seq3xpress, cells were resuspended in pure PBS to avoid serum contamination in the lysis mix.
Instrument	FACSAria III Cell Sorter system (BD Biosciences).
Software	FACS data was analyzed with FlowJo software v10.8.0.
Cell population abundance	The purity of the sorted populations was determined by re-analysis of the populations from a bulk sort subsequent to single cell sorting.
Gating strategy	We selected cells excluding debris, singlet discrimination was performed using plots for forward scatter (FSC-A versus FSC-H) dead cells were excluded by SYTOX Blue Dead Cell Stain (ThermoFisher Scientific, S34857).

☒ Tick this box to confirm that a figure exemplifying the gating strategy is provided in the Supplementary Information.
